# MS1 Peptide Ion Intensity Chromatograms in MS2 (SWATH) Data Independent Acquisitions. Improving Post Acquisition Analysis of Proteomic Experiments*[Fn FN2]

**DOI:** 10.1074/mcp.O115.048181

**Published:** 2015-05-17

**Authors:** Matthew J. Rardin, Birgit Schilling, Lin-Yang Cheng, Brendan X. MacLean, Dylan J. Sorensen, Alexandria K. Sahu, Michael J. MacCoss, Olga Vitek, Bradford W. Gibson

**Affiliations:** From the ‡Buck Institute for Research on Aging, 8001 Redwood Blvd, Novato, California 94945;; §Department of Statistics, Purdue University, West Lafayette, IN 47907;; ¶College of Science, College of Computer and Information Science, Northeastern University, Boston, Massachusetts 02115;; ‖Department of Genome Sciences, University of Washington School of Medicine, Seattle, Washington;; **Department of Pharmaceutical Chemistry, University of California, San Francisco, California 94143

## Abstract

Quantitative analysis of discovery-based proteomic workflows now relies on high-throughput large-scale methods for identification and quantitation of proteins and post-translational modifications. Advancements in label-free quantitative techniques, using either data-dependent or data-independent mass spectrometric acquisitions, have coincided with improved instrumentation featuring greater precision, increased mass accuracy, and faster scan speeds. We recently reported on a new quantitative method called MS1 Filtering (Schilling *et al.* (2012) *Mol. Cell. Proteomics* 11, 202–214) for processing data-independent MS1 ion intensity chromatograms from peptide analytes using the Skyline software platform. In contrast, data-independent acquisitions from MS2 scans, or SWATH, can quantify all fragment ion intensities when reference spectra are available. As each SWATH acquisition cycle typically contains an MS1 scan, these two independent label-free quantitative approaches can be acquired in a single experiment. Here, we have expanded the capability of Skyline to extract both MS1 and MS2 ion intensity chromatograms from a single SWATH data-independent acquisition in an Integrated Dual Scan Analysis approach. The performance of both MS1 and MS2 data was examined in simple and complex samples using standard concentration curves. Cases of interferences in MS1 and MS2 ion intensity data were assessed, as were the differentiation and quantitation of phosphopeptide isomers in MS2 scan data. In addition, we demonstrated an approach for optimization of SWATH *m*/*z* window sizes to reduce interferences using MS1 scans as a guide. Finally, a correlation analysis was performed on both MS1 and MS2 ion intensity data obtained from SWATH acquisitions on a complex mixture using a linear model that automatically removes signals containing interferences. This work demonstrates the practical advantages of properly acquiring and processing MS1 precursor data in addition to MS2 fragment ion intensity data in a data-independent acquisition (SWATH), and provides an approach to simultaneously obtain independent measurements of relative peptide abundance from a single experiment.

Mass spectrometry is the leading technology for large-scale identification and quantitation of proteins and post-translational modifications (PTMs)^1^ in biological systems (1, 2). Although several types of experimental designs are employed in such workflows, most large-scale applications use data-dependent acquisitions (DDA) where peptide precursors are first identified in the MS1 scan and one or more peaks are then selected for subsequent fragmentation to generate their corresponding MS2 spectra. In experiments using DDA, one can employ either chemical/metabolic labeling or label-free strategies for relative quantitation of peptides (and proteins) (3, 4). Depending on the type of labeling approach employed, *i.e.* metabolic labeling with SILAC or postmetabolic labeling with ICAT or isobaric tags such as iTRAQ or TMT, the relative quantitation of these peptides are made using either MS1 or MS2 ion intensity data (4–7). Label-free quantitative techniques have until recently been based entirely on integrated ion intensity measurements of precursors in the MS1 scan, or in the case of spectral counting the number of assigned MS2 spectra (3, 8, 9).

Label-free approaches have recently generated more widespread interest (10–12), in part because of their adaptability to a wide range of proteomic workflows, including human samples that are not amenable to most metabolic labeling techniques, or where chemical labeling may be cost prohibitive and/or interfere with subsequent enrichment steps (11, 13). However the use of DDA for label-free quantitation is also susceptible to several limitations including insufficient reproducibility because of under-sampling, digestion efficiency, as well as misidentifications (14, 15). Moreover, low ion abundance may prohibit peptide selection, especially in complex samples (14). These limitations often present challenges in data analysis when making comparisons across samples, or when a peptide is sampled in only one of the study conditions.

To address the challenges in obtaining more comprehensive sampling in MS1 space, Purvine *et al.* first demonstrated the ability to obtain sequence information from peptides fragmented across the entire *m*/*z* range using “shotgun or parallel collision-induced dissociation (CID)” on an orthogonal time of flight instrument (16). Shortly thereafter Venable *et al.* reported on a data independent acquisition methodology to limit the complexity of the MS2 scan by using a segmented approach for the sequential isolation and fragmentation of all peptides in a defined precursor window (*e.g.* 10 *m*/*z*) using an ion trap mass spectrometer (17). However, the proper implementation of this DIA technique suffered from technical limitations of instruments available at that time, including slow acquisition rates and low MS2 resolution that made systematic product ion extraction problematic. To alleviate the challenge of long duty cycles in DIAs, researchers at the Waters Corporation adopted an alternative approach by rapidly switching between low (MS1) and high energy (MS2) scans and then using proprietary software to align peptide precursor and fragment ion information to determine peptide sequences (18, 19). Recent mass spectrometry innovations in efficient high-speed scanning capabilities, together with high-resolution data acquisition of both MS1 and MS2 scans, and multiplexing of scan windows have overcome many of these limitations (10, 20, 21). Moreover, the simultaneous development of novel software solutions for extracting ion intensity chromatograms based on spectral libraries has enabled the use of DIA for large-scale label free quantitation of multiple peptide analytes (21, 22). In addition to targeting specific peptides from a previously generated peptide spectral library, the data can also be reexamined (*i.e.* post-acquisition) for additional peptides of interest as new reference data emerges. On the SCIEX TripleTOF 5600, a quadrupole orthogonal time-of-flight mass spectrometer, this technique has been optimized and extended to what is called ‘SWATH MS2′ based on a combination of new technical and software improvements (10, 22).

In a DIA experiment a MS1 survey scan is carried out across the mass range followed by a SWATH MS2 acquisition series, however the cycle time of the MS1 scan is dramatically shortened compared with DDA type experiments. The Q1 quadrupole is set to transmit a wider window, typically Δ25 *m*/*z*, to the collision cell in incremental steps over the full mass range. Therefore the MS/MS spectra produced during a SWATH MS2 acquisition are of much greater complexity as the MS/MS spectra are a composite of all fragment ions produced from peptide analytes with molecular ions within the selected MS1 *m*/*z* window. The cycle of data independent MS1 survey scans and SWATH MS2 scans is repeated throughout the entire LC-MS acquisition. Fragment ion information contained in these SWATH MS2 spectra can be used to uniquely identify specific peptides by comparisons to reference spectra or spectral libraries. Moreover, ion intensities of these fragment ions can also be used for quantitation. Although MS2 typically increases selectivity and reduces the chemical noise often observed in MS1 scans, quantifying peptides from SWATH MS2 scans can be problematic because of the presence of interferences in one or more fragment ions or decreased ion intensity of MS2 scans as compared with the MS1 precursor ion abundance.

To partially alleviate some of these limitations in SWATH MS2 scan quantitation it is potentially advantageous to exploit MS1 ion intensity data, which is acquired independently as part of each SWATH scan cycle. Recently, our laboratories and others have developed label free quantitation tools for data dependent acquisitions (11, 12, 23) using MS1 ion intensity data. For example, the MS1 Filtering algorithm uses expanded features in the open source software application Skyline (11, 24). Skyline MS1 Filtering processes precursor ion intensity chromatograms of peptide analytes from full scan mass spectral data acquired during data dependent acquisitions by LC MS/MS. New graphical tools were developed within Skyline to enable visual inspection and manual interrogation and integration of extracted ion chromatograms across multiple acquisitions. MS1 Filtering was subsequently shown to have excellent linear response across several orders of magnitude with limits of detection in the low attomole range (11). We, and others, have demonstrated the utility of this method for carrying out large-scale quantitation of peptide analytes across a range of applications (25–28). However, quantifying peptides based on MS1 precursor ion intensities can be compromised by a low signal-to-noise ratio. This is particularly the case when quantifying low abundance peptides in a complex sample where the MS1 ion “background” signal is high, or when chromatograms contain interferences, or partial overlap of multiple target precursor ions.

Currently MS1 scans are underutilized or even deemphasized by some vendors during DIA workflows. However, we believe an opportunity exists that would improve data-independent acquisitions (DIA) experiments by including MS1 ion intensity data in the final data processing of LC-MS/MS acquisitions. Therefore, to address this possibility, we have adapted Skyline to efficiently extract and process both precursor and product ion chromatograms for label free quantitation across multiple samples. The graphical tools and features originally developed for SRM and MS1 Filtering experiments have been expanded to process DIA data sets from multiple vendors including SCIEX, Thermo, Waters, Bruker, and Agilent. These expanded features provide a single platform for data mining of targeted proteomics using both the MS1 and MS2 scans that we call Integrated Dual Scan Analysis, or IDSA. As a test of this approach, a series of SWATH MS2 acquisitions of simple and complex mixtures was analyzed on an SCIEX TripleTOF 5600 mass spectrometer. We also investigated the use of MS2 scans for differentiating a case of phosphopeptide isomers that are indistinguishable at the MS1 level. In addition, we investigated whether smaller SWATH *m*/*z* windows would provide more reliable quantitative data in these cases by reducing the number of potential interferences. Lastly, we performed a statistical assessment of the accuracy and reproducibility of the estimated (log) fold change of mitochondrial lysates from mouse liver at different concentration levels to better assess the overall value of acquiring MS1 and MS2 data in combination and as independent measurements during DIA experiments.

## EXPERIMENTAL PROCEDURES

### 

#### 

##### Materials

HPLC solvents including acetonitrile and water were obtained from Burdick & Jackson (Muskegon, MI). Reagents for protein chemistry including iodoacetamide, dithiothreitol (DTT), ammonium bicarbonate, formic acid, trifluoroacetic acid, acetic acid, dichloroacetic acid (DCA), dodecyl-maltoside, and urea were purchased from Sigma Aldrich (St. Louis, MO). All protein standards were >95% purity. Tris(2-carboxyethyl)phosphine (TCEP) was purchased from Thermo (Rockford, IL), and HLB Oasis SPE cartridges were purchased from Waters (Milford, MA). Proteomics grade trypsin was from Promega (Madison WI). Trypsin-predigested beta-galactosidase (a quality control standard) was purchased from SCIEX (Foster City, CA).

##### Response Curves for a set of Acetylated Peptides in a Complex Matrix

Six lysine-acetylated synthetic peptides containing ^13^C_6_^15^N_2_-Lys and ^13^C_6_^15^N_4_-Arg were used to generate standard concentration curves in either a simple (25 fmol “six protein mix”) or a complex matrix (complex mitochondrial lysate, 0.3 μg on column), spanning from 4 attomoles to 25 femtomoles over 6 concentration points (0.004, 0.012, 0.037, 0.111, 0.333, 1, 3, and 25 fmol) for the following peptides: LVSSVSDLPKacR (HMGCS2 protein), MVQKacSLAR (HMGCS2 protein), AFVDSCLQLHETKacR (LCAD protein), YAPVAKacDLASR (SDHA protein), LFVDKacIR (ATP5J protein), and AFGGQSLKacFGK (SDHA protein). Three replicate concentration curves, each with injections from lowest to highest spike concentration were acquired on the TripleTOF 5600 (SWATH MS2 mode).

##### Mouse Liver Mitochondrial Protein Lysate

Mitochondria were isolated by differential centrifugation from liver WT (C57BL/6) mice, and proteins were denatured with 1% dodecyl-maltoside and 10 m urea. Samples were then diluted 1:10, reduced with 4.5 mm TCEP (37 °C for 1 h), alkylated with 10 mm iodoacetamide (30 min at RT in the dark), and incubated overnight at 37 °C with sequencing grade trypsin added at a 1:50 enzyme/substrate ratio (wt/wt). Samples were then acidified with formic acid and desalted using HLB Oasis SPE cartridges. Samples were eluted, concentrated to near dryness, and resuspended prior to analysis. Samples were processed in duplicates and three injection replicates at a concentration of 100 ng or 33 ng were acquired in a randomized order on the TripleTOF 5600 mass spectrometer either as is or spiked into an *E. coli* hydrolysate of 300 ng for additional complexity.

##### PHDE1α Kinase Inhibitor Study

Mouse liver mitochondria from wild-type mice (C57BL/6) were isolated as described previously (29, 30). Mitochondria (1 mg) were incubated at room temperature with 5 mm DCA (10 mm Hepes pH 7.2) for 0, 5, 10, 30, 60, and 120 min. Samples were digested with trypsin and phosphopeptides were enriched by IMAC chromatography as previously described (11). Heavy phosphopeptides for pSer-293 (YHGHS^293^MSDPGVSYR[^13^C_6_^15^N_4_]) and pSer-300 (YHGHSMSDPGVS^300^YR[^13^C_6_^15^N_4_]) were spiked at 25 fmol into each sample. Equal volumes of eluted phosphopeptides were desalted using C-18 zip-tips and then analyzed on the TripleTOF 5600.

##### Mass Spectrometry

Mass spectrometric data was acquired on a quadrupole time-of-flight (QqTOF) TripleTOF 5600 (SCIEX, Concord, Canada) directly connected to a reverse-phase HPLC-ESI-MS/MS using an Eksigent Ultra Plus nano-LC 2D HPLC system (Dublin, CA). Samples were acquired in data dependent acquisitions (DDA) and in data independent acquisitions (DIA) SWATH MS2 acquisition modes. In the SWATH MS2 acquisitions, the Q1 quadrupole transmits a wider window of ∼25 *m*/*z* in incremental steps over the full mass range (*m*/*z* 400–1000) in 24 SWATH segments. In addition, different size SWATH windows, *i.e.* at 10, 12.5, and 6.25 *m*/*z* were acquired with adjustments in accumulation times or total covered MS1 *m*/*z* range to compensate for the increased number of Δ *m*/*z* segments. Detailed descriptions of MS and HPLC parameters and conditions are provided in the Supplemental Methods S1.

##### Database Searches

Mass spectral data sets were analyzed and searched using the database search engine ProteinPilot (31) (SCIEX Beta 4.1.46, revision 460) using the Paragon algorithm (4.0.0.0, 459). The following sample parameters were used: trypsin digestion, cysteine alkylation set to carbamidomethylation and species *M. musculus*. Processing parameters were set to “Biological modification” and a thorough ID search effort was used. More detailed descriptions can be found in the supplemental Methods S1.

##### Quantitative MS1 and SWATH MS2 Data Analysis in Skyline

MS1 and MS2 chromatogram based quantitation was carried out in Skyline 2.5 and 2.6 (24) an open source software project (http://proteome.gs.washington.edu/software/skyline) as recently described in detail for MS1 Filtering (11). First, comprehensive spectral libraries were generated in Skyline from database searches of the raw data files prior to MS1 Filtering. Second, all raw files acquired in DDA, were directly imported into Skyline and MS1 precursor ions were extracted for all peptides present in the MS/MS spectral libraries. Quantitative MS1 analysis was based on extracted ion chromatograms (XICs) and for the top 3 resulting precursor ion peak areas *e.g.* M, M+1, and M+2. Final quantitative comparisons were typically based on only the highest ranked precursor ion. In contrast, SWATH MS2 data sets are targeted DIA assays, and Skyline quantitation was based on XICs of up to 10 MS/MS fragment ions, typically y- and b-ions, matching to specific peptides present in the spectral libraries described above.

##### Statistical Analysis Mitochondrial Lysates from Mouse Liver

Mitochondrial samples were prepared with concentrations at 100 ng and 33 ng on column, or spiked into an *E. coli* lysate of 300 ng for a total of 400 ng and 333 ng on column. Data was acquired on the TripleTOF 5600 from 2 process and 3 injection replicates and then analyzed as follows. First, because both MS1 and MS2 scans are subject to quantitative interferences and noise, we determined an initial representative quantitative profile of each peptide based on area under the curve (AUC) of XIC peaks. The coefficient of variation (CV) for the peptide precursor was calculated using the most abundant isotopic peak across all replicates, whereas the CV for the MS2 scan was calculated using the average log intensity of the 3 most intense MS2 fragment ions across all runs. If a peptide had a lower CV for the MS1 peaks, we retained the MS1 peaks as the representative quantitative profile. Otherwise, we retained the average log-intensity of the 3 most intense MS2 peaks as the representative quantitative profile. Second, we used the representative profiles to select other informative MS1 and/or MS2 peaks, and to remove noisy peaks. We calculated, separately for each peptide, the Pearson coefficient of correlation between each MS1 and MS2 extracted ion and the representative profile above over all runs. Most of the coefficients of correlation were relatively high. In particular, 90% of the correlation values were above 0.95 for the lysate without the *E. coli* background, and above 0.73 for the lysate with the *E. coli* background. However, there were a few low-correlating outliers. To separate the majority of highly correlated ions from the outliers we defined a correlation cutoff, and only kept the ions with the correlation above the cutoff for the subsequent statistical analysis. The cutoff was defined as the smallest percentile of all the correlations in the experiment, such that it was within 3% of the next smallest percentile. For the lysate without the *E. coli* background the cutoff corresponded to the 10th percentile (correlation 0.95), and we removed 10% of the ions with correlation below this value. For the lysate with the *E. coli* background the cutoff corresponded to the 30th percentile (correlation 0.94), and we removed 30% of the ions with correlations below this value.

In order to assess the accuracy of the estimation of log2 fold change and the ability to detect the change in abundance, we specified an additive linear fixed effects model, which is an instance of the model implemented in MSstats (32, 33). The model has been optimized to assess the value of MS1 and MS2 data for a controlled mixture using the specified data set and will need to be further refined for use with biological data sets where additional biological variation is common. Specifically, the model is:

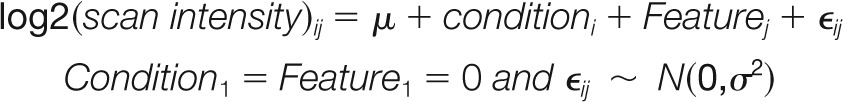
 where *i* = 1,2 is the index of the mixture, and *j* is the index of the MS1 or MS2 peaks selected in the procedure above. In this model, the parameter *Condition*_2_ is the estimate of log2 fold-change between the mixtures. The fold change on the original scale is then obtained as Condition_2_. The test for differential abundance corresponds to the null hypothesis H_0_: *Condition*_2_ = 0. Finally, because the samples were analyzed at concentrations of 100 ng and 33 ng, all the peptides in this study are differentially abundant. Therefore, an additional analysis was performed to assess the specificity of the results. We randomly assigned 3 samples from mixture 1 to Group 1 and the other 3 samples from mixture 1 to Group 2, and the same model was fit to this new randomized data set.

The results of these analyses were compared with the results of the same modeling and testing procedure, but applied to all the MS2 peaks or all the MS1 peaks. Note that when the procedure was applied to the MS2 peaks, the average log-intensity of the top-3 MS2 fragments was used as the representative profile for all peptides.

##### Data Accession

All mass spectrometry data has been uploaded to the Center for Computational Mass Spectrometry under the “Mass spectrometry Interactive Virtual Environment,” MassIVE, and can be downloaded using the following ftp link: ftp://massive.ucsd.edu/MSV000079092 (username: MSV000079092). The MS1 MS2 SWATH data sets and Skyline files uploaded to Panorama can be accessed at https://panoramaweb.org/labkey/MS1MS2.url.

## RESULTS AND DISCUSSION

### 

#### 

##### MS Acquisition and Processing of Data Independent MS1 and MS2 Scans in Skyline

**Fig. 1.**
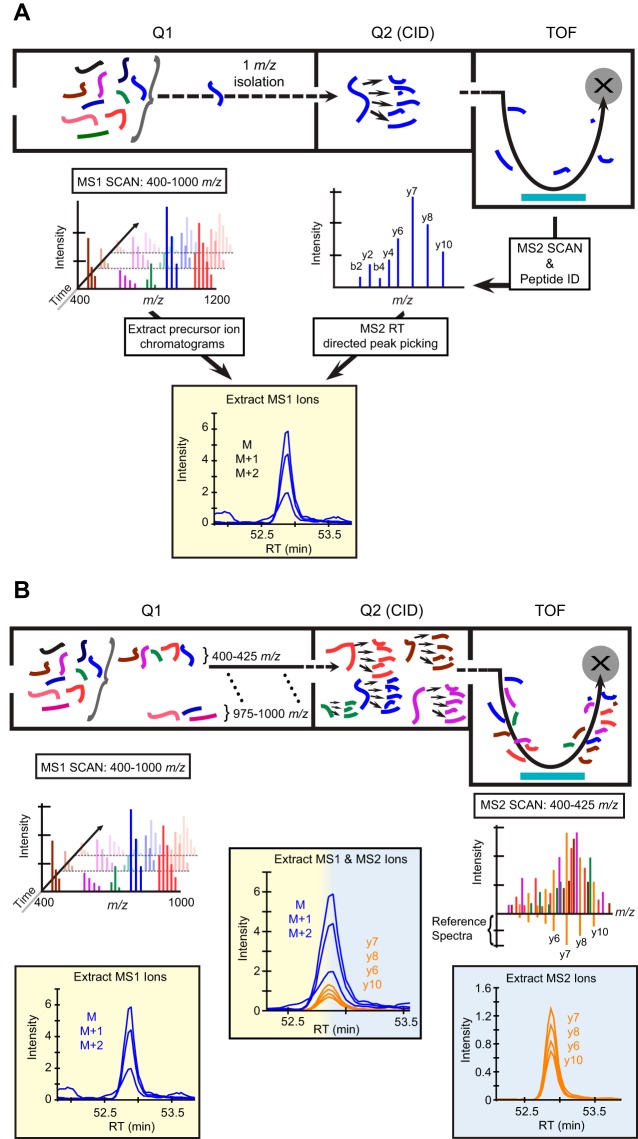
**Schematic for data dependent and data independent workflows using a QqTOF.** *A*, During a high-resolution data dependent acquisition, an MS1 scan (400–1200 *m*/*z*) of the precursor ions is performed in the first quadrupole (Q1). Ion abundances above a set threshold are selectively collided with nitrogen in Q2 generating an MS2 scan of the fragment ions. MS1 Filtering in Skyline extracts peptide precursor ion intensity chromatograms from the MS1 scan for a given precursor isotopic envelope (M, M+1, M+2, etc.). Retention time of the corresponding peptide identified from underlying acquired MS/MS directs proper selection and integration of the precursor ion peak used for relative quantitation. *B*, During data independent acquisitions quantitative data can be extracted for a given peptide precursor ion from the MS1 scan using MS1 Filtering (yellow), and simultaneously from the MS2 scans (blue) acquired as stepwise SWATH acquisitions selecting user defined precursor ion mass range windows (*e.g.* 400–425 *m*/*z*, 425–450 *m*/*z*, etc.). The complex MS/MS spectra can later be deconvoluted using a peptide spectral library and fragment ion intensity chromatograms can be extracted for quantitation.

MS1 Filtering in Skyline (11) is typically employed to process full scan ion intensity chromatograms from data dependent acquisitions (Fig. 1*A*). In proteomic DDA methodology on QqTOF instruments, an MS1 survey scan (*e.g.* 400–1200 *m*/*z*) is performed in Q1 to determine the ion abundance of precursor analytes entering the mass spectrometer. Peptide precursor signals above the MS/MS threshold are selected and transmitted through a narrow 1 *m*/*z* window into the collision cell (Q2) for tandem mass spectrometry. MS/MS fragment ions from individually selected peptides are detected in the TOF detector at high resolution, and are classically used for database searches and subsequently for peptide sequence information. Following a database search, peptides with high confidence MS/MS spectra are converted into spectral libraries within Skyline. MS1 Filtering matches the retention time of the peptide identification information, to automatically integrate MS1 scan chromatograms from the raw data corresponding to a given peptide sequence within Skyline. In addition, MS1 Filtering extracts the isotopic envelope (M, M+1, M+2, etc.) (Fig. 1*A*) from the MS1 scan and scores it against the theoretical isotopic distribution. However, during data-independent acquisitions (referred to as SWATH) quantitative data can be extracted for a given peptide precursor ion from the MS1 scan using MS1 Filtering (yellow, left), and simultaneously for fragment ions from the MS2 scans (blue, right). MS2 scans were acquired as “looped” MS/MS spectra obtained from SWATH segments transmitting MS1 mass range windows (*e.g.* 400–425 *m*/*z*, 425–450, etc.) rather than specific peptide precursor ions (Fig. 1*B*). Subsequently, spectral libraries originating from traditional DDA database searches are used to extract fragment ion intensity information. We adapted Skyline to extract both MS1 and MS2 transitions (precursor and fragment ions) in a simultaneous workflow, which can be interrogated in a single user interface window (Fig. 1*B*).

##### Comparison of MS1 and MS2 Quantitation using Standard Response Curves

**Fig. 2.**
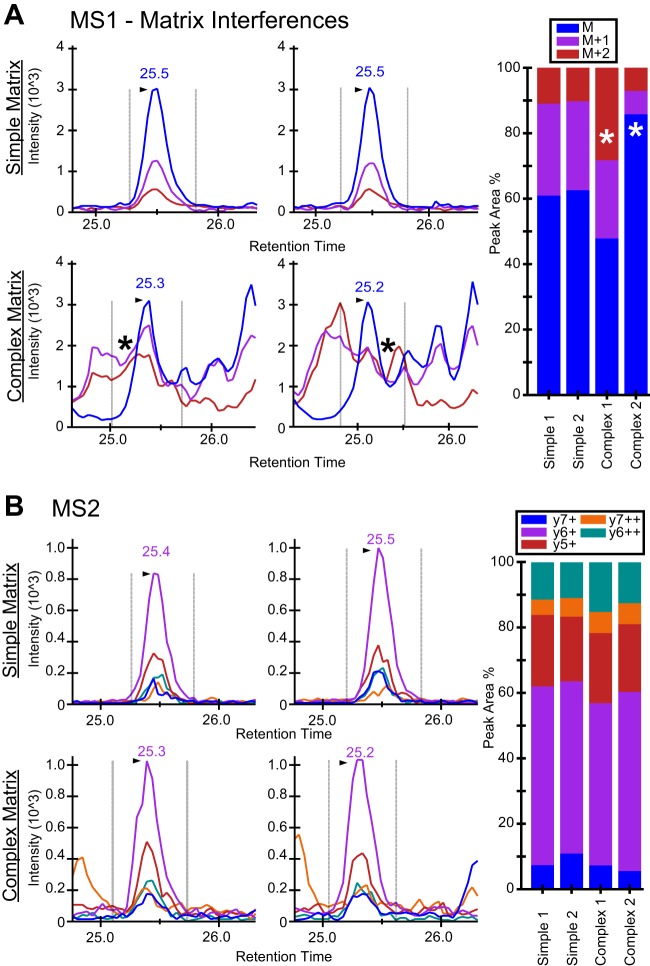
**Precursor ion interferences observed during MS1 Filtering.** *A*, Interferences observed in MS1 Filtered peak area intensity traces for the precursor ion *m*/*z* 492.78^2+^ (heavy MVQ**Kac**SLAR[^13^C_6_^15^N_4_]) spiked at 300 amol into either a simple (top panel, 25 fmol “six protein mix”) or complex matrix (bottom panel, mitochondrial lysate from mouse liver, 0.3 μg on column) and acquired on a TripleTOF 5600 mass spectrometer in triplicates. MS1 precursor ion peak areas (M, M+1, M+2) as a percent of the total area are displayed in the bar graph along with the expected or theoretical ion distribution. *B*, Corresponding MS2 fragment ion intensity traces extracted from the same acquisitions as in *A* for both simple (top panel) and complex (bottom panel) matrixes show no interferences. MS2 fragment ion peak areas as a percent of the total area are displayed in the bar graph along with the expected or theoretical ion distribution.

To compare the robustness and linear response of MS1 and MS2 chromatogram extraction from SWATH acquisitions, we carried out a series of dilution experiments. Heavy isotope labeled peptides ^13^C_6_,^15^N_2_-lysine or ^13^C_6_,^15^N_4_–arginine were spiked into a simple matrix (commercial 6-protein mix) or complex matrix (300 ng mouse liver mitochondrial lysate) spanning a concentration range from 4 attomoles to 25 fmoles. Although both the MS1 and MS2 chromatogram peaks had strong linearity across several orders of magnitude in the simple matrix (supplemental Fig. S1*A*), the MS1 peak had less variability at lower concentrations. Peptides spiked into the complex matrix also displayed strong linearity in both MS1 and MS2 at higher concentrations. However, the MS1 signal appeared to be less robust at the lower concentrations as compared with MS2 (supplemental Fig. S1*B*). The MS1 precursor ions appeared to be more prone to encounter interferences whereas the more selective MS2 fragment ions typically had fewer interferences and thus achieved a better linear response and better dynamic range in the complex matrix (supplemental Fig. S1*B*). This can be readily visualized when analyzing multiple acquisitions of the stable isotope labeled peptide (MVQKacetylSLA**R,** where ***R*** = ^13^C_6_,^15^N_4_-Arg) where 300 attomoles was spiked into a simple matrix consisting of 25 fmol of a six protein mix or the complex matrix consisting of 300 ng of a mouse liver mitochondrial lysate (Fig. 2*A*). The upper panel displays a strong and easily identifiable peak in the simple matrix. The lower panel shows widespread interferences in proper peak selection and integration, which leads to increased variability in the data. However, the corresponding SWATH MS2 XICs display no evidence of interferences in either the simple or the complex matrix, likely because of the higher selectivity on the MS2 scan level (Fig. 2*B*).

##### Differentiating and Quantitation of Co-eluting Phosphopeptide Isomers in MS2 Scans

Accurate assignment of PTMs remains a significant challenge when multiple sites are present within a given peptide (34, 35). In particular, phosphopeptide isomers with the same *precursor mass* typically have very similar chromatographic properties when the phosphorylation site varies between Ser, Thr, and/or Tyr residues within the same peptide. Co-eluting peptide isomers may also yield chimeric spectra that are difficult to interpret. In addition, dynamic exclusion (a common technique used to limit repetitive sampling in discovery workflows) may miss peptide isomers with small chromatographic shifts. To further explore the potential benefits of SWATH for quantifying phosphopeptide isomers, we carried out a study using mouse liver mitochondria treated with DCA, an inhibitor of the pyruvate dehydrogenase kinases.

**Fig. 3.**
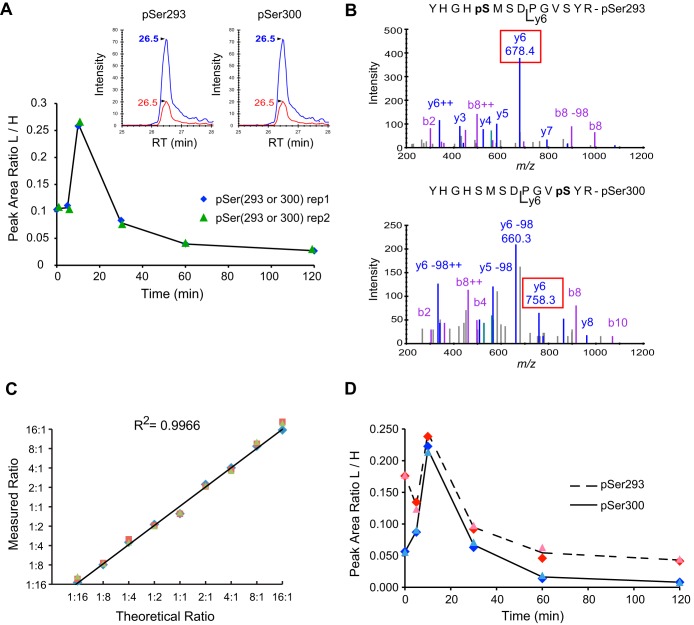
**Quantitation of mono-phosphopeptide isomers across time following kinase inhibition with DCA using SWATH acquisitions.** *A*, Relative quantitation (MS1 Filtering) of endogenous mono phosphopeptides YHGH**pS**^293^MSDPGVSYR and YHGHSMSDPGV**pS**^300^YR with precursor ion M extracted at *m*/*z* 558.22^3+^ following DCA time course treatment. Inset shows precursor ion traces for both the light endogenous (red trace) and heavy (blue trace), spiked in (Arg is ^13^C_6_^15^N_4_) phosphopeptide isomers at the 10 min time point. *B*, MS/MS spectra for endogenous pSer293 (*m*/*z* 558.2^3+^, top panel) and pSer300 (*m*/*z* 558.22^3+^, bottom panel) with distinguishing fragment ions used for quantitation boxed in red. *C*, Synthetic phosphopeptide isomers with the phosphorylation site at either pSer293 or pSer300 were spiked into a six-protein mix at ratios ranging from 1:16 to 16:1 in twofold intervals. Observed peak area ratios from SWATH MS2 scans were formed by calculating the ratio between pSer293/pSer300 from peak areas from corresponding fragment ions: y_6_/[y_6_ + (y_6_-98)]. *D*, SWATH MS2 quantitation for changes in phosphorylation at Ser293, and Ser300 in PDHE1α following kinase inhibition with DCA. Isotopically labeled synthetic phosphopeptides YHGH**pS**^293^MSDPGVSYR[^13^C_6_^15^N_4_] and YHGHSMSDPGV**pS**^300^Y R[^13^C_6_^15^N_4_] were spiked each at 25 fmol. Fragment ion XICs of [y6] and [y6 + (y6–98)] pairs, respectively, were used to form ratios to the corresponding heavy peptide isomers.

In pyruvate dehydrogenase E1α (PDHE1α), two well-characterized phosphorylation sites are present within the same tryptic peptide at pSer-293 (YHGHS^293^MSDPGVSYR) or at pSer-300 (YHGHSMSDPGVS^300^YR) (30). As these phosphopeptide isomers co-elute under the separation conditions used here, they are indistinguishable when analyzed by MS1 Filtering, even in the presence of heavy isotope labeled phosphopeptide standards (Fig. 3*A*). However, synthetic stable isotope peptides of the corresponding phospho isomers revealed that these isomers could be differentiated by their MS2 fragment ions; the y_6_ ion at *m*/*z* 678.4 originates from the pSer293 isomer, whereas the y_6_/y_6_-98 ion pair at *m*/*z* 758.3/660.3 results from fragment ions of the pSer300 isomer (Fig. 3*B*). To demonstrate that SWATH could be used to quantitate and differentiate phosphopeptide isomers, the heavy peptides YHGHpS^293^MSDPGVSY**R** and YHGHSMSDPGVpS^300^Y**R** (where ***R*** = ^13^C_6_,^15^N_4_-Arg) were spiked into 25 fmol of a six-protein mix at ratios ranging from 1:16 to 16:1 in twofold intervals and analyzed in triplicate. The y_6 (_pSer-293) and the y_6_ + y_6_-98 (pSer-300) ion pair ratios had strong linearity (R^2^ - 0.9966) across the differentially spiked in samples (Fig. 3*C*). Details regarding the isomer response curve are shown in supplemental Fig. S2. Finally, to demonstrate that SWATH could be used to differentiate and quantify biologically relevant samples, we monitored changes in phosphorylation at Ser293 and Ser300 of endogenous PDHE1α following kinase inhibition with DCA. Heavy synthetic phosphopeptides corresponding to pSer293 and pSer300 were spiked into tryptic digested mitochondrial lysates at 25 fmol each prior to phosphopeptide affinity enrichments (IMAC) and MS analysis. During SWATH data processing fragment ion XICs of [y_6_] and [y_6_ + (y_6_-98)] pairs, respectively, were used to determine relative ratios of the corresponding heavy peptide isomers. MS2 based analysis revealed that with kinase inhibition, the pS293 site was dephosphorylated slightly faster and to a slightly larger extent compared with pS300. Taken together this demonstrates that SWATH can be used to differentiate and quantitate co-eluting peptide isomers that are indistinguishable at the MS1 level.

##### Identifying Interferences in SWATH Quantitative Workflows Using MS1 Scans

**Fig. 4.**
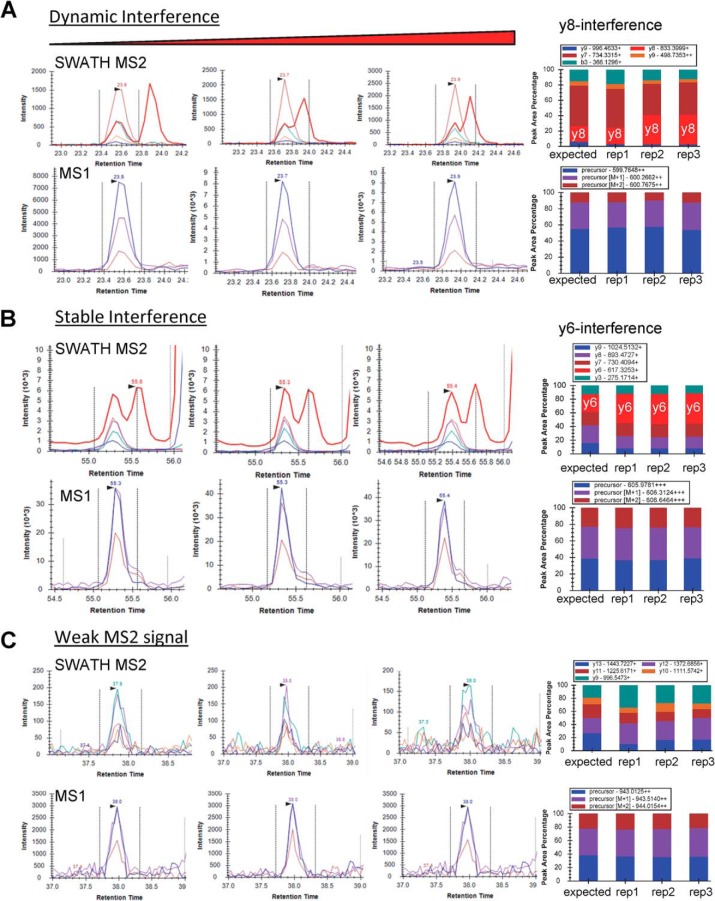
**Different types of MS2 fragment ion interferences observed during SWATH acquisition.** *A, Dynamic or sporadic interference* of the y8 fragment ion (red trace) obtained from the precursor ion *m*/*z* - 599.76^2+^ (DSYVGDEAQSK) observed across multiple acquisitions of the same peptide. MS1 Filtering results for the same peptide are shown directly below with highly reproducible MS1 peak areas across all replicates. MS2 fragment ion peak areas as a percent of the total area are displayed in the bar graph along with the expected or theoretical ion distribution. *B, Stable interference* of the y6 fragment ion (red trace) obtained from the precursor ion *m/z* at 605.9^3+^ (TTSLELFMYLNEVAGK) across 3 acquisitions for the same peptide. *C*, Overall weak MS2 signal obtained from the ion *m/z* - 943.01^2+^ (KVITAFNDGLNHLDSLK). MS2 fragment ions show significant interferences from background noise across three acquisitions.

Although there are clear advantages to utilizing the MS2 scan for carrying out SWATH quantitative workflows, we also examined the additional value of extracting the MS1 scan data acquired as part of the SWATH MS2 scan cycle. In support of using MS1 data, we observed that in some cases the SWATH MS2 scans had more interferences than the MS1 precursor ion data across replicates. Fig. 4*A* shows one such case, called “dynamic” or sporadic interference, which occurred when an unrelated fragment ion interference transitions across the peak of interest over multiple replicates leading to an erroneous peak area determination in an abundant y-ion fragment ion. This is likely because of small variations in chromatographic properties between the interference and the peptide of interest and would lead to decreased reproducibility of the measurement. Alternatively, a second type of interference, labeled as a “stable” interference (Fig. 4*B*), where a MS2 interference in the y-ion fragment appeared in all replicates with a similar aberrant elution profile and would lead to an inaccurate measure of the peptide abundance. Last, Fig. 4*C* shows an example where the MS2 fragment ions were all of very low abundance and were impacted by interferences originating from background noise across the acquisitions. In all three cases, a clearly abundant MS1 signal showed no interferences, thus the MS1 ion chromatograms could alert one to a potential MS2 summing error.

##### Reduction of MS2 Scan Interferences in SWATH using MS1 Scan Optimization

**Fig. 5.**
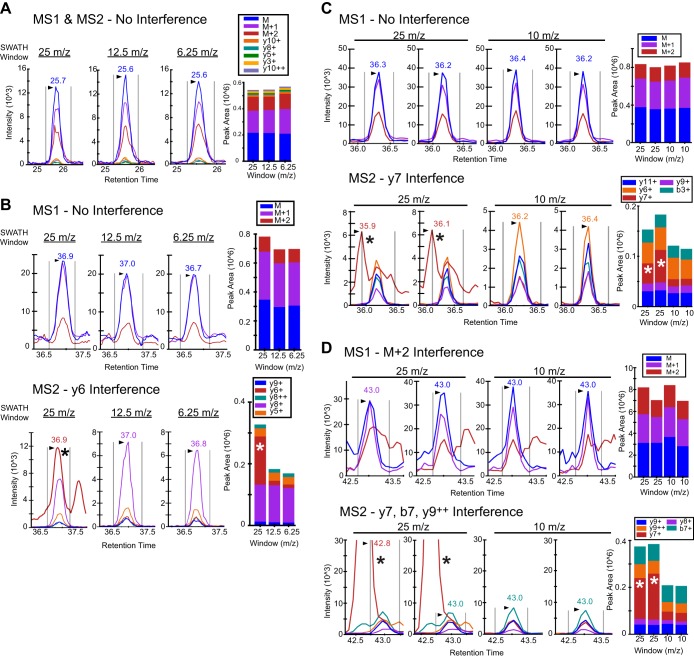
**Optimization of SWATH acquisition window width using MS1 Filtering.** *A*, Peak area traces and bar graphs from MS1 and MS2 scan data obtained from the precursor ion *m*/*z* at 716.32^2+^ (YMCENQATISSK) from a mitochondrial lysate acquired in triplicates using SWATH acquisition windows of 25, 12.5, and 6.25 *m*/*z*, respectively. Precursor and fragment ions are displayed together. *B*, Peak area traces and bar graphs from MS1 (top panel) and MS2 (bottom panel) data obtained from the precursor ion *m*/*z* at 701.86^2+^ (EEIIPVAPEYDK) from a mitochondrial lysate acquired in triplicates using SWATH acquisition windows of 25, 12.5, and 6.25 *m*/*z*, respectively. The y_6_^+^ fragment ion interference is indicated with an asterisk in the MS2 trace bar plot from the 25 *m/z* SWATH window acquisition. *C*, Peak area traces and bar graphs from MS1 and MS2 data obtained from the precursor ion *m*/*z* at 745.87^2+^ (GILAADESVGTMGNR) from a mitochondrial lysate acquired with five replicates each using SWATH acquisition windows of 25 and 10 *m/z*, respectively. The y_7_^+^ fragment ion interference is indicated with an asterisk in the MS2 trace bar plot from the 25 *m*/*z* SWATH window acquisition. *D*, Peak area traces and bar graphs from MS1 (top panel) and MS2 (bottom panel) data obtained from the precursor ion *m*/*z* at 594.34^3+^ (RTGAIVDVPVGEELLGR) from a mitochondrial lysate acquired with 5 replicates each using SWATH acquisition windows of 25 and 10 *m*/*z*, respectively. The MS2 chromatograms below indicate interferences for the y_7_^+^, b_7_^+^, and y_9_^2+^ fragment ions as indicated with asterisks in the MS2 trace bar plot from the 25 *m*/*z* SWATH window acquisition.

Since the implementation of SWATH MS2 and similar DIA methods into proteomic workflows, we and others (21) have investigated ways to optimize SWATH acquisitions, including the *m*/*z* segment width of the SWATH windows. Alterations in segment width were therefore examined, with the goal to achieve optimal transition selectivity while maintaining sufficient accumulation and cycle times. It is worth mentioning that SWATH acquisition windows can be adapted according to the specific requirements of the project, where one would make adjustments best suited for the complexity of the expected sample type. For example, Fig. 5*A* and 5*B* display MS1 and MS2 extracted ion chromatograms from complex mitochondrial lysates at SWATH segment widths of 25, 12.5, and 6.25 *m*/*z*. Although Panel A shows no interferences, Panel B shows clean MS1 scans but interferences in the MS2 space (y_6_^+^ ion) at 25 *m*/*z* segment width, which, however, decreases and is eliminated when smaller SWATH segment widths are used. As shown here, the MS1 signal can help to identify MS2 interferences. Similar examples are shown in Fig. 5*C* and 5*D*, where four mitochondrial lysate replicates, each with SWATH acquisition windows of 25 and 10 *m*/*z*, respectively, show MS2 interferences in the 25 *m*/*z* window but not in the more narrow 10 *m*/*z* window in two separate peptides. The reduction of interferences using smaller SWATH windows will of course be sample dependent, however ideally the instrument would adjust to the complexity of *m*/*z* space by automatically varying the segment width in response to this complexity. For example, the instrument would use a smaller segment of 4 *m*/*z* when analyzing average peptides (*e.g.* 500–600 *m*/*z*) and a larger segment window of 50 *m*/*z* when sampling larger peptides (1000–1100 *m*/*z*). This concept of SWATH variable window width will be explored in future studies.

In addition to cases where a decrease in the width of the SWATH window can reduce interferences in the MS2 fragment ions, we were also interested in the effect on the reproducibility of these measurements. To assess this, we examined five separate acquisitions of mitochondrial lysates with SWATH segment window widths of 25 *m*/*z* and 10 *m*/*z*, respectively, to generate scatter plots of peak area coefficient of variation (CV). In these comparisons, we observed that the number of individual MS2 outlier transitions with higher peak area CV's tend to be slightly increased in the 25 *m*/*z* segment width acquisitions, as compared with the 10 *m*/*z* segment width data sets (supplemental Fig. S3*A*). Not surprisingly, the effect is more evident for the least abundant peptides. However, the majority of the data shows very good reproducibility with CV's less than 20%. The contribution of individual nonrobust outlier transitions can be attenuated when calculating peptide peak areas as the sum of the top five fragment ion peak areas per peptide (supplemental Fig. S3*B*). However, statistical methods can also filter out unreliable fragment ions automatically, as demonstrated below.

##### Interference Reduction using a Linear Fixed Effects Model

**Fig. 6.**
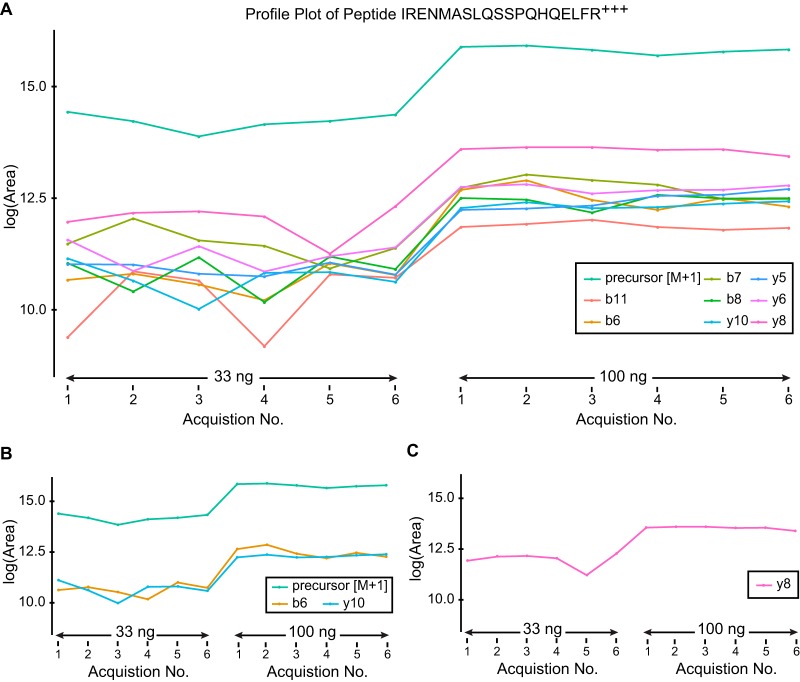
**Enhanced interference reduction using a linear fixed effects model with IDSA.** *A*, Representative profile plot for peptide area for IRENMASLQSSPQHQELFR (757.71^3+^ *m*/*z*) from ACADV (very long-chain specific acyl-CoA dehydrogenase) across all acquisitions. Represents one of the 100 peptides, where the CV of the rank-one MS1 scan was smaller than the average of the top-3 MS2 scans from 283 peptides. *B*, The same peptide as in *A* following interference removal using MS1 scan information to guide feature selection. *C*, Profile plot using MS2 to guide feature selection when MS1 signal is ignored or not acquired. Considering the correlation of all the scans with respect to the top-3 MS2 scans would result in incorrectly selected scans.

To investigate the potential benefit of using an integrated dual scan analysis (IDSA) of both MS1 and MS2 scans to guide feature selection for removal of interferences, we analyzed the data set of a mitochondrial lysate without a background matrix at a predetermined ratio of 3:1 (supplemental Table S1) as described in the Experimental Methods. Of the 283 peptides we assessed, 100 had a lower CV across acquisitions using the MS1 precursor ion as compared with that of the average log-intensity for the top 3 MS2 fragment ions, and the MS1 precursor ion was retained as the representative profile. For the remaining 183 peptides, the average log-intensity of the top 3 MS2 fragment ions had a lower CV, and this was retained as the representative profile. Noisy scans were then filtered out as described in the Experimental Methods. When MS1 scans were utilized in addition to MS2 scans, the filtering procedure resulted in 3–9 acceptable chromatogram peaks per peptide, and the MS1 precursor peaks for all 283 peptides were retained as part of the representative peptide profiles indicating the quality of the MS1 derived data is acceptable. Fig. 6*A* describes a peptide whose MS1 precursor ion had a lower CV than that of the average log-intensity of the top3 MS2 fragment ions. In this example using MS1 precursor ion data as the representative profile provided a better filter (Fig. 6*B*) rather than using the average log-intensity of the top 3 MS2 fragment ions (Fig. 6*C*). This demonstrates a potential advantage to acquiring MS1 scans in addition to MS2 scans for enhanced feature selection and reduction of inferences.

**Fig. 7.**
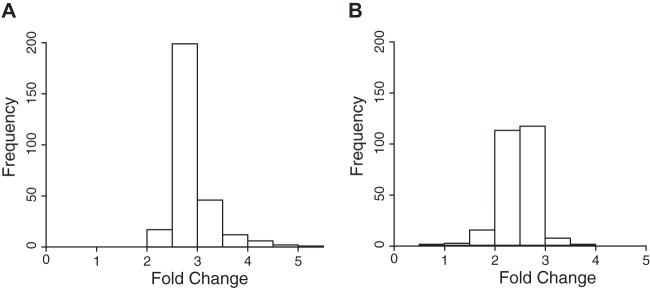
**Distribution of the fold change using the joint analysis of MS1 and MS2 scans.** *A*, Distribution of the fold change measured across 283 peptides with no background, or *B*, in the presence of an *E. coli* background matrix of 300 ng. The true fold change is 3 in both *A* and *B*.

To compare the effectiveness of our linear model using the dual scan analysis (MS1 and MS2), we considered the distribution of the log-fold changes across all 283 peptides (Fig. 7*A*). Our results showed that the estimates are centered around the true log-fold change, however the distribution is slightly skewed. Finally, to further compare the effectiveness of the refined selection of the fragments, we performed an independent estimation of the accuracy to the true fold change using a joint analysis as compared with using individual MS1 or MS2 ion chromatograms (Table I). Our results demonstrate that the removal of interferences, regardless of scan type, is beneficial for both MS1 and MS2 ion intensity data, and each of the three methods (IDSA, MS2 only, and MS1 only) estimated the fold change similarly well (Table I). The improved overall estimation of the true fold change is modest, however this would be expected in a large data set where a minority of peptides would be subject to interferences. In addition the inter-quartile range is improved when interferences are filtered out for all cases demonstrating reduced variability and improved reproducibility across measurements (Table I).

**Table I:** Summary of the estimated fold change. Fold Change = 2 (model-based estimate of log[Fold Change])

	Filtered	Min	Q1	Median	Q3	Max	Interquartile range
No background	*MS1 and MS2*	2.232	2.619	2.790	2.935	5.524	0.316
	*MS2 only*	2.205	2.606	2.772	2.926	5.526	0.32
	*MS1 only*	2.232	2.607	2.789	2.935	5.524	0.328

	Unfiltered	Min	Q1	Median	Q3	Max	Interquartile range
No background	*MS1 and MS2*	2.145	2.619	2.788	2.966	5.525	0.347
	*MS2 only*	1.988	2.583	2.755	2.926	5.526	0.343
	*MS1 only*	2.083	2.729	2.867	3.048	5.505	0.319

	Filtered	*^a^*Min	Q1	Median	Q3	*^b^*Max	Interquartile range
*E. coli* background	*MS1 and MS2*	1.412	2.296	2.428	2.581	3.024	0.285
	*MS2 only*	1.854	2.254	2.415	2.566	3.054	0.312
	*MS1 only*	1.068	2.257	2.419	2.563	3.400	0.306

	Unfiltered	*^a^*Min	Q1	Median	Q3	*^b^*Max	Interquartile range
*E. coli* background	*MS1 and MS2*	1.437	2.134	2.333	2.503	3.015	0.369
	*MS2 only*	1.408	2.085	2.325	2.500	3.037	0.415
	*MS1 only*	1.068	2.225	2.529	2.734	3.602	0.509

*^a^* Excludes bottom 1%.

*^b^* Excludes top 1%.

To further evaluate our model in a more complex biological background where the vast majority of matrix peptides would be unchanged, we examined the same mitochondrial lysate as described above at the same 3:1 ratio, but where it was now spiked into a complex *E. coli* background matrix, (*i.e.* 33 ng or 100 ng added to 300 ng of a total *E. coli* protein hydrolysate). Of the 283 peptides originally quantified 22 were considered unquantifiable in the *E. coli* background as all ions had a CV > 30%. From the remaining 261 peptides, 68 had a lower CV in the MS1 signal as compared with the average of the top 3 fragment ions. In addition, this new analysis revealed a greater level of suppression in the true fold change (2.428) as compared with the mitochondrial lysate without background (2.790) (Fig. 7*A* and 7*B*). This is likely because of ion suppression from the increase in both the chemical and peptide noise present within the *E. coli* matrix. However, the use of combined MS1 and MS2 data for removal of interferences provided an even more significant improvement in the measure of the true fold change, and with better reproducibility, when compared with using either data set individually (Table I). In addition, we did not observe a dependence on the peptide abundance for measuring the fold change (Fig. S4). It should be pointed out that the differences between the three methods (MS1, MS2, IDSA) may be more pronounced in other experimental regimens, where the true fold changes are less well defined than in these experiments. In any case our results demonstrate that if the MS1 data is acquired appropriately when using a DIA approach this information may be used to remove interferences from complex samples to yield more accurate and reproducible data sets.

## CONCLUSION

The use of data-independent acquisition (DIA) methodologies such as SWATH has recently garnered significant interest from the proteomics community for use in quantitative workflows. This type of acquisition has several desirable properties, including the ability to quantify as many peptides as are typically identified using DDA, strong reproducibility, a large dynamic range, and the ability to revisit the data post acquisition for new targets (10). As quantitation in DIA is carried out using ion intensities of MS2 fragments, the role of MS1 precursors ions in these workflows have largely been ignored, despite the fact that their respective ion abundances are higher. In our case a high resolution MS1 scan of good quality requires <8% of the total DIA duty cycle, making its acquisition time minimally disruptive for the majority of experiments. We have described a new approach developed as part of the Skyline software platform for extraction of ion intensity measurements from MS1 and MS2 scans, called Integrated Dual Scan Analysis (IDSA) that has several distinct advantages. Importantly, both MS1 and MS2 data sets can be extracted and analyzed from a single acquisition in combination or individually. Moreover, we show that this data can be used to better detect MS2 ion interferences, combined for downstream statistical analysis, help assign peptide isomers, and be used independently for validation of MS2-based quantitation.

When investigating standard concentration curves in simple and complex matrix, both quantitation methods SWATH-MS2 and MS1 Filtering showed strong reproducibility, linear responses across several orders of magnitude, and LOQs into the low attomole range (60–185 amol) with CVs typically below 20%. Although MS1 Filtering showed a slight advantage in simple matrix, possibly because of higher ion abundance of the MS1 signal without significant interferences, SWATH MS2 acquisition performed better in more complex matrices. The better performance of MS2 in complex mixtures is not unexpected (10), as this is likely because of the higher selectivity in extracting fragment ions and lower signal background. However, in most cases MS1 and MS2 quantitation methods correlated well and provided similar results.

Another clear advantage to IDSA is the ability to simultaneously quantify MS1 and SWATH-MS2 to reduce false peak integrations caused by interferences and/or weak signals. In fact, cases of ion interferences can be more readily identified in IDSA as MS1 and MS2 integrations would not be expected to yield the same result. Inconsistencies between MS1 and MS2 abundance measurements can therefore be easily flagged and manually interrogated if so desired. Overall, the reduction and/or elimination of interferences are essential to ensure robust and reproducible quantitative results. Interestingly, some analytical tasks cannot be achieved using MS1 scan quantitation, such as in the case of distinguishing ion intensity contributions from co-eluting PTM-peptide isomers. SWATH MS2 quantitation, however, was clearly capable of differentiating between two phosphosite isomers once unique fragment ions were assigned to each isomer. Although we acknowledge that site assignment could be differentiated by SRM using peptide standards, the time required for developing such assays is not practical in the majority of cases when analyzing large discovery-type data sets, thus making SWATH MS2 a viable option for differentiating and quantifying co-eluting PTM isomers.

The focus of the current study was carried out using a 5600 TripleTOF from Sciex using SWATH for DIA. However, we should point out that other instrument manufacturers, some with quite different sector and ion optic designs (*e.g.* Orbitrap, FT-MS), have also begun to develop and implement quantitative DIA methodologies. Many of these DIA methods, like SWATH, also acquire a high resolution MS1 scan as part of their acquisition cycle, although it is not clear to what extent, if any, MS1 ion intensity data is being used in peptide quantitation. Previous work explored the correlation of peptide precursor and fragment ion data using Waters unique LC-MS^E^ approach and indicated the potential use of both scans for quantitation purposes (36). Based on this previous study (36) and the work described here, we believe that including MS1 ion intensity data in a DIA acquisition is well worth the small price in duty cycle, and could become a critical part of DIA-based quantitation methods as well as providing an independent means for validating changes in peptides concentrations. Overall, the parallel acquisition of different scan types can be used synergistically. Each quantitated MS1 signal should always show co-eluting MS2 fragment ions, thus confirming peptide identities in each replicate independent of sampling. Similarly, MS2 fragment ions can be correlated to their precursor ions that are simultaneously sampled with MS1 scans, and in most cases providing independent confirmation of their identities.

Lastly, when we first began this investigation, we were limited by the available DIA software for the selection of fragment ions used for final ion intensity summations. These algorithms would typically not filter out contaminated fragment ions, thus leading to clear and not infrequent cases of inaccurate peptide abundance determinations using MS2 fragments ions. However, as the study progressed, we utilized a linear modeling framework for both MS1 and MS2 ion intensity data that helped to automatically eliminate these interferences and improve the estimation of fold changes. Data processed using this optimized interference filtering method now shows that one can essentially derive correct assessments of peptides abundance changes across samples in a SWATH experiment whether one uses MS1, MS2, or combined MS1/MS2 data (IDSA). Based on the level of sample complexity, there will be cases where one of these three approaches may be preferred. Overall this work demonstrates the practical advantages to acquiring MS1 precursor abundances as part of IDSA with minimal loss to overall cycle time during the MS2 scan allowing one to generate combined quantitative data. In addition, previous work demonstrated the feasibility of using DIAs to independently confirm MS1 ion intensity measurements from DDAs (37); however, our current results demonstrate that if the MS1 data is acquired appropriately when using a DIA approach it may be used as an independent validation of fragment ion abundances acquired during a single acquisition. As DIA methods are rapidly evolving, both in terms of the instrumentation and software, we believe this work provides a framework to consider the utility of including MS1 ion intensity data, and suggests that ultimate effectiveness of doing so may need to be assessed on a case-by-case basis.

## Supplementary Material

Supplemental Data

## Footnotes

^1^ The abbreviations used are:

PTM: post translational modification
DIA: data independent acquisition
DDA: data dependent acquisition
IDSA: Integrated Dual Scan Analysis
LC-MS: liquid chromatography mass spectrometry
MS/MS: tandem mass spectrometry
QqTOF: quadrupole-quadrupole-time-of-flight
MS1: full scan mass spectrum
MS2: fragment ion scan mass spectrum
M: monoisotopic mass
SRM: single reaction monitoring
DTT: dithiothreitol
TCEP: tris(2-carboxyethyl)phosphine
DCA: dichloroacetic acid
SPE: solid phase extraction
ACN: acetonitrile
XIC: extracted ion chromatogram
FDR: false discovery rate
CV: coefficient of variation
PHDE1α: pyruvate dehydrogenase E1 alpha
SDHA: succinate dehydrogenase subunit A
HMGCS2: hydroxymethylglutaryl CoA synthase 2
ATP5J: ATP synthase-coupling factor 6
LCAD: long chain acyl-CoA dehydrogenase
ACADV: very long-chain specific acyl-CoA dehydrogenase
IMAC: immobilized metal affinity chromatography.

## References

[1] Olsen J. V. Mann M. (2013) Status of large-scale analysis of post-translational modifications by mass spectrometry. Mol. Cell. Proteomics 12, 3444–3452 24187339 10.1074/mcp.O113.034181 PMC3861698

[2] Bantscheff M. Lemeer S. Savitski M. M. Kuster B. (2012) Quantitative mass spectrometry in proteomics: critical review update from 2007 to the present. Anal. Bioanal. Chem. 404, 939–965 22772140 10.1007/s00216-012-6203-4

[3] Neilson K. A. Ali N. A. Muralidharan S. Mirzaei M. Mariani M. Assadourian G. Lee A. van Sluyter S. C. Haynes P. A. (2011) Less label, more free: approaches in label-free quantitative mass spectrometry. Proteomics 11, 535–553 21243637 10.1002/pmic.201000553

[4] Ross P. L. Huang Y. N. Marchese J. N. Williamson B. Parker K. Hattan S. Khainovski N. Pillai S. Dey S. Daniels S. Purkayastha S. Juhasz P. Martin S. Bartlet-Jones M. He F. Jacobson A. Pappin D. J. (2004) Multiplexed protein quantitation in Saccharomyces cerevisiae using amine-reactive isobaric tagging reagents. Mol. Cell. Proteomics 3, 1154–1169 15385600 10.1074/mcp.M400129-MCP200

[5] Ong S. E. Blagoev B. Kratchmarova I. Kristensen D. B. Steen H. Pandey A. Mann M. (2002) Stable isotope labeling by amino acids in cell culture, SILAC, as a simple and accurate approach to expression proteomics. Mol. Cell. Proteomics 1, 376–386 12118079 10.1074/mcp.m200025-mcp200

[6] Gygi S. P. Rist B. Gerber S. A. Turecek F. Gelb M. H. Aebersold R. (1999) Quantitative analysis of complex protein mixtures using isotope-coded affinity tags. Nat. Biotechnol. 17, 994–999 10504701 10.1038/13690

[7] Thompson A. Schafer J. Kuhn K. Kienle S. Schwarz J. Schmidt G. Neumann T. Johnstone R. Mohammed A. K. Hamon C. (2003) Tandem mass tags: a novel quantification strategy for comparative analysis of complex protein mixtures by MS/MS. Anal. Chem. 75, 1895–1904 12713048 10.1021/ac0262560

[8] Ishihama Y. Oda Y. Tabata T. Sato T. Nagasu T. Rappsilber J. Mann M. (2005) Exponentially modified protein abundance index (emPAI) for estimation of absolute protein amount in proteomics by the number of sequenced peptides per protein. Mol. Cell. Proteomics 4, 1265–1272 15958392 10.1074/mcp.M500061-MCP200

[9] Liu H. Sadygov R. G. Yates J. R. 3rd (2004) A model for random sampling and estimation of relative protein abundance in shotgun proteomics. Anal. Chem. 76, 4193–4201 15253663 10.1021/ac0498563

[10] Gillet L. C. Navarro P. Tate S. Rost H. Selevsek N. Reiter L. Bonner R. Aebersold R. (2012) Targeted data extraction of the MS/MS spectra generated by data-independent acquisition: a new concept for consistent and accurate proteome analysis. Mol. Cell. Proteomics 11, O111.016717 10.1074/mcp.O111.016717 PMC3433915 22261725

[11] Schilling B. Rardin M. J. MacLean B. X. Zawadzka A. M. Frewen B. E. Cusack M. P. Sorensen D. J. Bereman M. S. Jing E. Wu C. C. Verdin E. Kahn C. R. Maccoss M. J. Gibson B. W. (2012) Platform-independent and label-free quantitation of proteomic data using MS1 extracted ion chromatograms in skyline: application to protein acetylation and phosphorylation. Mol. Cell. Proteomics 11, 202–214 22454539 10.1074/mcp.M112.017707 PMC3418851

[12] Cox J. Hein M. Y. Luber C. A. Paron I. Nagaraj N. Mann M. (2014) Accurate proteome-wide label-free quantification by delayed normalization and maximal peptide ratio extraction, termed MaxLFQ. Mol. Cell. Proteomics 13, 2513–2526 24942700 10.1074/mcp.M113.031591 PMC4159666

[13] Qian W. J. Jacobs J. M. Liu T. Camp D. G. 2nd Smith R. D. (2006) Advances and challenges in liquid chromatography-mass spectrometry-based proteomics profiling for clinical applications. Mol. Cell. Proteomics 5, 1727–1744 16887931 10.1074/mcp.M600162-MCP200 PMC1781927

[14] Tabb D. L. Vega-Montoto L. Rudnick P. A. Variyath A. M. Ham A. J. Bunk D. M. Kilpatrick L. E. Billheimer D. D. Blackman R. K. Cardasis H. L. Carr S. A. Clauser K. R. Jaffe J. D. Kowalski K. A. Neubert T. A. Regnier F. E. Schilling B. Tegeler T. J. Wang M. Wang P. Whiteaker J. R. Zimmerman L. J. Fisher S. J. Gibson B. W. Kinsinger C. R. Mesri M. Rodriguez H. Stein S. E. Tempst P. Paulovich A. G. Liebler D. C. Spiegelman C. (2010) Repeatability and reproducibility in proteomic identifications by liquid chromatography-tandem mass spectrometry. J. Proteome Res. 9, 761–776 19921851 10.1021/pr9006365 PMC2818771

[15] Bell A. W. Deutsch E. W. Au C. E. Kearney R. E. Beavis R. Sechi S. Nilsson T. Bergeron J. J. Group H. T. S. W. (2009) A HUPO test sample study reveals common problems in mass spectrometry-based proteomics. Nat. Methods 6, 423–430 19448641 10.1038/nmeth.1333 PMC2785450

[16] Purvine S. Eppel J. T. Yi E. C. Goodlett D. R. (2003) Shotgun collision-induced dissociation of peptides using a time of flight mass analyzer. Proteomics 3, 847–850 12833507 10.1002/pmic.200300362

[17] Venable J. D. Dong M. Q. Wohlschlegel J. Dillin A. Yates J. R. (2004) Automated approach for quantitative analysis of complex peptide mixtures from tandem mass spectra. Nat. Methods 1, 39–45 15782151 10.1038/nmeth705

[18] Silva J. C. Denny R. Dorschel C. A. Gorenstein M. Kass I. J. Li G. Z. McKenna T. Nold M. J. Richardson K. Young P. Geromanos S. (2005) Quantitative proteomic analysis by accurate mass retention time pairs. Anal. Chem. 77, 2187–2200 15801753 10.1021/ac048455k

[19] Silva J. C. Gorenstein M. V. Li G. Z. Vissers J. P. Geromanos S. J. (2006) Absolute quantification of proteins by LCMSE: a virtue of parallel MS acquisition. Mol. Cell. Proteomics 5, 144–156 16219938 10.1074/mcp.M500230-MCP200

[20] Panchaud A. Scherl A. Shaffer S. A. von Haller P. D. Kulasekara H. D. Miller S. I. Goodlett D. R. (2009) Precursor acquisition independent from ion count: how to dive deeper into the proteomics ocean. Anal. Chem. 81, 6481–6488 19572557 10.1021/ac900888s PMC3086478

[21] Egertson J. D. Kuehn A. Merrihew G. E. Bateman N. W. MacLean B. X. Ting Y. S. Canterbury J. D. Marsh D. M. Kellmann M. Zabrouskov V. Wu C. C. MacCoss M. J. (2013) Multiplexed MS/MS for improved data-independent acquisition. Nat. Methods 10, 744–746 23793237 10.1038/nmeth.2528 PMC3881977

[22] Rost H. L. Rosenberger G. Navarro P. Gillet L. Miladinovic S. M. Schubert O. T. Wolski W. Collins B. C. Malmstrom J. Malmstrom L. Aebersold R. (2014) OpenSWATH enables automated, targeted analysis of data-independent acquisition MS data. Nat. Biotechnol. 32, 219–223 24727770 10.1038/nbt.2841

[23] Sandin M. Teleman J. Malmstrom J. Levander F. (2014) Data processing methods and quality control strategies for label-free LC-MS protein quantification. Biochim. Biophys. Acta 1844, 29–41 23567904 10.1016/j.bbapap.2013.03.026

[24] MacLean B. Tomazela D. M. Shulman N. Chambers M. Finney G. L. Frewen B. Kern R. Tabb D. L. Liebler D. C. MacCoss M. J. (2010) Skyline: an open source document editor for creating and analyzing targeted proteomics experiments. Bioinformatics 26, 966–968 20147306 10.1093/bioinformatics/btq054 PMC2844992

[25] Guo A. Gu H. Zhou J. Mulhern D. Wang Y. Lee K. A. Yang V. Aguiar M. Kornhauser J. Jia X. Ren J. Beausoleil S. A. Silva J. C. Vemulapalli V. Bedford M. T. Comb M. J. (2014) Immunoaffinity enrichment and mass spectrometry analysis of protein methylation. Mol. Cell. Proteomics 13, 372–387 24129315 10.1074/mcp.O113.027870 PMC3879628

[26] Rardin M. J. He W. Nishida Y. Newman J. C. Carrico C. Danielson S. R. Guo A. Gut P. Sahu A. K. Li B. Uppala R. Fitch M. Riiff T. Zhu L. Zhou J. Mulhern D. Stevens R. D. Ilkayeva O. R. Newgard C. B. Jacobson M. P. Hellerstein M. Goetzman E. S. Gibson B. W. Verdin E. (2013) SIRT5 regulates the mitochondrial lysine succinylome and metabolic networks. Cell Metab. 18, 920–933 24315375 10.1016/j.cmet.2013.11.013 PMC4105152

[27] Rardin M. J. Newman J. C. Held J. M. Cusack M. P. Sorensen D. J. Li B. Schilling B. Mooney S. D. Kahn C. R. Verdin E. Gibson B. W. (2013) Label-free quantitative proteomics of the lysine acetylome in mitochondria identifies substrates of SIRT3 in metabolic pathways. Proc. Natl. Acad. Sci. U.S.A. 110, 6601–6606 23576753 10.1073/pnas.1302961110 PMC3631688

[28] Sos M. L. Levin R. S. Gordan J. D. Oses-Prieto J. A. Webber J. T. Salt M. Hann B. Burlingame A. L. McCormick F. Bandyopadhyay S. Shokat K. M. (2014) Oncogene mimicry as a mechanism of primary resistance to BRAF inhibitors. Cell Reports 8, 1037–1048 25127139 10.1016/j.celrep.2014.07.010 PMC4294625

[29] Rardin M. J. Wiley S. E. Murphy A. N. Pagliarini D. J. Dixon J. E. (2008) Dual specificity phosphatases 18 and 21 target to opposing sides of the mitochondrial inner membrane. J. Biol. Chem. 283, 15440–15450 18385140 10.1074/jbc.M709547200 PMC2397459

[30] Rardin M. J. Wiley S. E. Naviaux R. K. Murphy A. N. Dixon J. E. (2009) Monitoring phosphorylation of the pyruvate dehydrogenase complex. Anal. Biochem. 389, 157–164 19341700 10.1016/j.ab.2009.03.040 PMC2713743

[31] Shilov I. V. Seymour S. L. Patel A. A. Loboda A. Tang W. H. Keating S. P. Hunter C. L. Nuwaysir L. M. Schaeffer D. A. (2007) The Paragon Algorithm, a next generation search engine that uses sequence temperature values and feature probabilities to identify peptides from tandem mass spectra. Mol. Cell. Proteomics 6, 1638–1655 17533153 10.1074/mcp.T600050-MCP200

[32] Clough T. Key M. Ott I. Ragg S. Schadow G. Vitek O. (2009) Protein quantification in label-free LC-MS experiments. J. Proteome Res. 8, 5275–5284 19891509 10.1021/pr900610q

[33] Clough T. Thaminy S. Ragg S. Aebersold R. Vitek O. (2012) Statistical protein quantification and significance analysis in label-free LC-MS experiments with complex designs. BMC Bioinformatics 13, S6 23176351 10.1186/1471-2105-13-S16-S6 PMC3489535

[34] Beausoleil S. A. Villen J. Gerber S. A. Rush J. Gygi S. P. (2006) A probability-based approach for high-throughput protein phosphorylation analysis and site localization. Nat. Biotechnol. 24, 1285–1292 16964243 10.1038/nbt1240

[35] Savitski M. M. Lemeer S. Boesche M. Lang M. Mathieson T. Bantscheff M. Kuster B. (2011) Confident phosphorylation site localization using the Mascot Delta Score. Mol. Cell. Proteomics 10, M110 003830 10.1074/mcp.M110.003830 PMC3033680 21057138

[36] Geromanos S. J. Vissers J. P. Silva J. C. Dorschel C. A. Li G. Z. Gorenstein M. V. Bateman R. H. Langridge J. I. (2009) The detection, correlation, and comparison of peptide precursor and product ions from data independent LC-MS with data dependant LC-MS/MS. Proteomics 9, 1683–1695 19294628 10.1002/pmic.200800562

[37] Kuhn M. L. Zemaitaitis B. Hu L. I. Sahu A. Sorensen D. Minasov G. Lima B. P. Scholle M. Mrksich M. Anderson W. F. Gibson B. W. Schilling B. Wolfe A. J. (2014) Structural, kinetic and proteomic characterization of acetyl phosphate-dependent bacterial protein acetylation. PLoS One 9, e94816 24756028 10.1371/journal.pone.0094816 PMC3995681

