# Supplementary material for: MS1 Peptide Ion Intensity Chromatograms in MS2 (SWATH) Data Independent Acquisitions. Improving Post Acquisition Analysis of Proteomic Experiments
Source: Mol Cell Proteomics. 2015 May 17;14(9):2405–19. doi: 10.1074/mcp.O115.048181 (PMC4563724; doi:10.1074/mcp.O115.048181)
Supplement: Supplemental Data [file supp_14_9_2405__index.html]

MS1 Peptide Ion Intensity Chromatograms in MS2 (SWATH) Data Independent Acquisitions. Improving Post Acquisition Analysis of Proteomic Experiments. — MS1 Peptide Ion Intensity Chromatograms in MS2 (SWATH) Data Independent Acquisitions. Improving Post Acquisition Analysis of Proteomic Experiments — MS1 and MS2 Crosstalk in Data Independent Acquisitions — Supplemental Data 

# MS1 Peptide Ion Intensity Chromatograms in MS2 (SWATH) Data Independent Acquisitions. Improving Post Acquisition Analysis of Proteomic Experiments

## Supplemental Data

- Supplemental Methods S1 - Supplemental Methods S1 - Nano-LC-MS/MS analyses and Bioinformatic searches
- Supplemental Table S1 - Supplemental Table S1 - S1A. Skyline Quantitative output for precursor and fragment ions of samples : 100 ng and 33 ng mitochondrial lysates, 2 process and 3 technical replicates each; S1B. Protein Pilot Database search results of mouse mitochondrial protein lysates underlying Skyline spectral libraries; S1C. Peptide precursor and fragment ion information for MS1MS2 quantiation after festure selection without background; S1D. Peptide precursor and fragment ion information for MS1MS2 quantitation after festure selection with E. coli background.
- Supplemental Figure S1 - Supplemental Figure S1 - Comparison of MS1 Filtering and SWATH Quantitation using standard concentration curves.
- Supplemental Figure S2 - Supplemental Figure S2 - SWATH Phospho Isomer differentiation at different ratios across the isomer response curve.
- Supplemental Figure S3 - Supplemental Figure S3 - SWATH reproducibility for MS2 fragment ions using SWATH segment window widths of 25 m/z and 10 m/z, respectively.
- Supplemental Figure S4 - Supplemental Figure S4 - Comparison of peptide abundance in the 100 ng mitochondrial lysate to fold change after feature selection using IDSA.
