# Supplementary material for: MS1 Peptide Ion Intensity Chromatograms in MS2 (SWATH) Data Independent Acquisitions. Improving Post Acquisition Analysis of Proteomic Experiments
Source: Mol Cell Proteomics. 2015 May 17;14(9):2405–19. doi: 10.1074/mcp.O115.048181 (PMC4563724; doi:10.1074/mcp.O115.048181)
Supplement: Supplemental Data [file supp_O115.048181_mcp.O115.048181-1.pdf]

**Supplemental Methods S1:**

*Mass spectrometric and chromatographic methods and instrumentation –*

AB SCIEX TripleTOF 5600: Samples were analyzed by reverse-phase HPLC-ESI-MS/MS using an Eksigent Ultra Plus nano-LC 2D HPLC system (Dublin, CA) which was directly connected to an AB SCIEX quadrupole time-of-flight (QqTOF) TripleTOF 5600 mass spectrometer (AB SCIEX, Concord, CAN) in direct injection mode. The autosampler was operated in full injection mode overfilling a 1 µl loop with 3 µl analyte for optimal sample delivery reproducibility. Briefly, after injection, peptide mixtures were transferred onto the analytical C18-nanocapillary HPLC column (C18 Acclaim PepMap100, 75 µm I.D. x 15 cm, 3 µm particle size, 100 Å pore size, Dionex, Sunnyvale, CA) and eluted at a flow rate of 300 nL/min using the following gradient: 3% solvent B in A (from 0-13 min), 3-7% solvent B in A (from 13-16 min), 7-25% solvent B in A (from 16-48 min), 25-40% solvent B in A (from 48-65 min), 40-90% solvent B in A (from 65-75 min) and 90% solvent B in A (from 75-85 min), with a total runtime of 120 min including mobile phase equilibration. Solvents were prepared as follows: mobile phase A: 2% acetonitrile/98% of 0.1% formic acid (v/v) in water, and mobile phase B: 98% acetonitrile/2% of 0.1% formic acid (v/v) in water. Mass spectra and tandem mass spectra were recorded in positive-ion and "high-sensitivity" mode with a resolution of ~35,000 full-width half-maximum. The nanospray needle voltage was typically 2,400 V in HPLC-MS mode. After acquisition of ~ 3 samples, TOF MS spectra and TOF MS/MS spectra were automatically calibrated during dynamic LC-MS & MS/MS autocalibration acquisitions injecting 25 fmol beta-galactosidase. Two different mass spectrometric acquisition workflows were performed in this study: **1) Data dependent acquisitions (DDA) on the TripleTOF 5600**: for collision induced dissociation tandem mass spectrometry (CID-MS/MS), the mass window for

precursor ion selection of the quadrupole mass analyzer was set to ± 1 *m/z*. The precursor ions were fragmented in a collision cell using nitrogen as the collision gas. Data dependent acquisition mode (DDA) was applied on the TripleTOF 5600 to obtain MS/MS spectra for the 30 most abundant precursor ions following each survey MS1 scan (250 msec acquisition time per MS1 scan, and typically 50 msec acquisition time per each MS/MS) yielding a cycle time of 1.75 sec. Dynamic exclusion features were based on value M not *m/z* and were set to an exclusion mass width 50 mDa and an exclusion duration of 15-20 sec. **2) Data independent acquisitions (DIA) on the TripleTOF 5600 - SWATH MS2 acquisitions.** In the SWATH MS2 acquisition, instead of the Q1 quadrupole transmitting a narrow mass range through to the collision cell, a wider window of ~25 *m/z* is passed in incremental steps over the full mass range (*m/z* 400-1000 with 24 SWATH segments, 80 msec accumulation time each, yielding a cycle time of 2.25 sec which includes one MS1 scan with 250 msec accumulation time). SWATH MS2 produces complex MS/MS spectra which are a composite of all the analytes within each selected Q1 *m/z* window. Additional SWATH acquisitions with different SWATH window (segment) sizes were also performed with window widths of i) 10 Da (mass range from *m/z* 400-1000 with SWATH segments, 50 msec accumulation time each, 250 msec MS1 scan, yielding a total cycle time of 3.25 sec), ii) window widths of 12.5 Da (mass range from *m/z* 500-800 with 24 SWATH segments, 80 msec accumulation time each, 250 msec MS1 scan, yielding a total cycle time of 2.25 sec), and iii) window widths of 6.25 Da (mass range from *m/z* 500-800 with 48 SWATH segments, 62.5 msec accumulation time each, 250 msec MS1 scan, yielding a total cycle time of 3.25 sec).

*Bioinformatic database searches for TripleTOF 5600* - In this study, mass spectral data sets were analyzed and searched using the database search engine ProteinPilot (1)(AB SCIEX Beta 4.1.46, revision 460) using the Paragon algorithm (4.0.0.0, 459). The following sample parameters were used: trypsin digestion, cysteine alkylation set to carbamidomethylation and species *M. musculus*. Trypsin enzyme was the default settings. Processing parameters were set to "Biological modification" and a thorough ID search effort was used. During the search, Protein Pilot performs an automatic mass recalibration of the data sets based on highly confident peptide spectra. Specifically, a first search iteration is done to select high confidence peptide identifications that are used to recalibrate both the MS and MS/MS data, which is automatically re-searched. All data files were searched using the SwissProt 2014_05 (May 2014) with a total of 33338 mouse protein sequences). For Protein Pilot Searches, to assess and restrict rates of false positive peptide/protein identifications, we used the Proteomics System Performance Evaluation Pipeline (PSPEP) tool available in ProteinPilot 4.1 beta. This tool automatically creates a concatenated forward and reverse decoy database, and provides an Excel output of the experimentally determined false discovery rate at the spectral, peptide and protein levels with standard statistical errors (1). The discriminating variable for the Paragon Algorithm is the peptide confidence value, which is a 0-99 scaled real number (1). For database searches, a cut-off peptide confidence value of 99 was chosen with the following justification. For searching the databases the Protein Pilot false discovery rate (FDR) analysis tool (PSPEP) algorithm (1) provided, at a minimum, a global FDR of 1% and a local FDR at 5% in all cases.

Supplemental References

1. Shilov, I. V., Seymour, S. L., Patel, A. A., Loboda, A., Tang, W. H., Keating, S. P., Hunter, C. L., Nuwaysir, L. M., and Schaeffer, D. A. (2007) The Paragon Algorithm, a next generation search engine that uses sequence temperature values and feature probabilities to identify peptides from tandem mass spectra. *Mol Cell Proteomics* 6, 1638-1655
