# Supplementary material for: MS1 Peptide Ion Intensity Chromatograms in MS2 (SWATH) Data Independent Acquisitions. Improving Post Acquisition Analysis of Proteomic Experiments
Source: Mol Cell Proteomics. 2015 May 17;14(9):2405–19. doi: 10.1074/mcp.O115.048181 (PMC4563724; doi:10.1074/mcp.O115.048181)
Supplement: Supplemental Data [file supp_O115.048181_mcp.O115.048181-3.pdf]

Supplemental Fig. S1

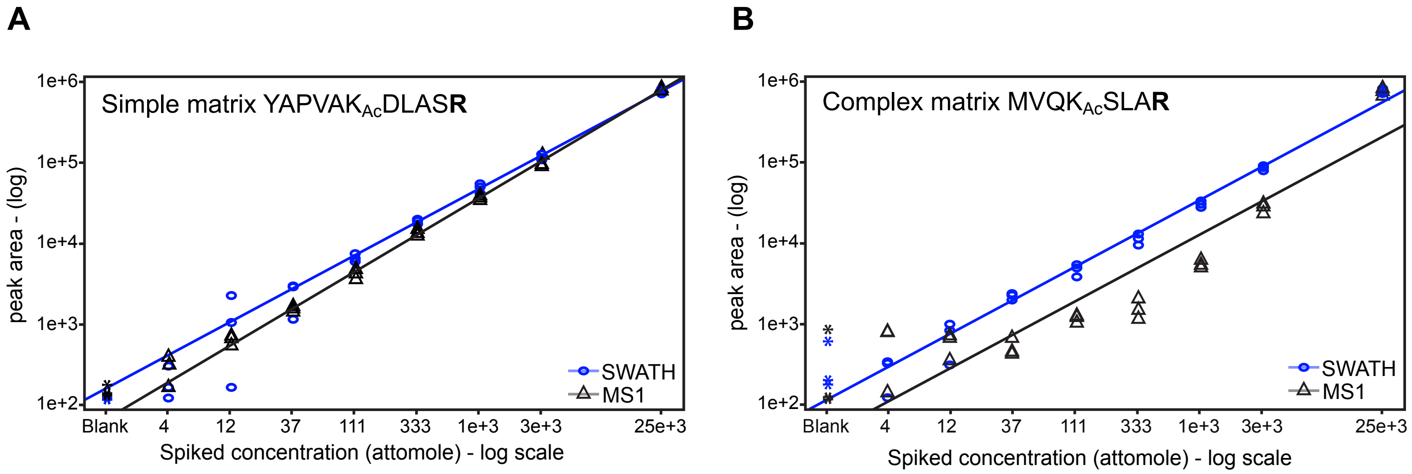

Supplemental Fig. S1. Comparison of MS1 Filtering and SWATH Quantitation using standard concentration curves and mitochondrial lysates. *A*, YAPVAKacDLASR[13C6 15N4]) (Kac is acetyllysine) , and *B*, MVQKacSLAR[$^{13}C_6$ $^{15}N_4$] spanning a concentration range from 4 amol to 25 fmol. Peptides were diluted into either a simple matrix (A, 25 fmol "six protein mix") or a complex matrix (B, 0.3 mg mitochondrial lysate from mouse liver), and acquired in triplicates. Simultaneous quantitation was carried out for MS1 and SWATH MS2 scans.
