# Supplementary material for: MS1 Peptide Ion Intensity Chromatograms in MS2 (SWATH) Data Independent Acquisitions. Improving Post Acquisition Analysis of Proteomic Experiments
Source: Mol Cell Proteomics. 2015 May 17;14(9):2405–19. doi: 10.1074/mcp.O115.048181 (PMC4563724; doi:10.1074/mcp.O115.048181)
Supplement: Supplemental Data [file supp_O115.048181_mcp.O115.048181-4.pdf]

Supplemental Fig. S2

A SWATH Phospho-Isomer differentiation – ratios pS293/ pS300 + 1:1

YHGH**pS**MSDPGVSY**R**

pS-293

y6 - 688.3652+ (heavy)

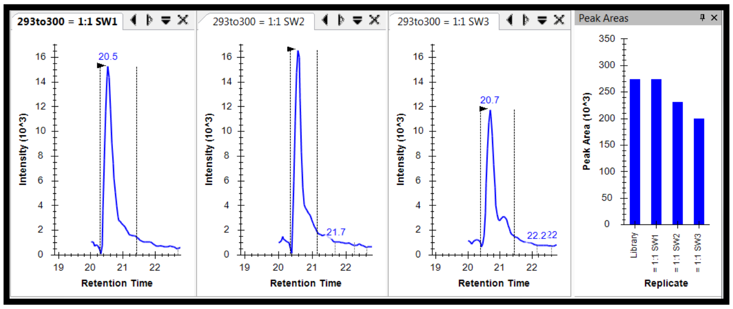

YHGHSMSDPGV**pS**YR

pS-300

y6 - 768.3316+ (heavy)
y6 -98 - 670.3547+ (heavy)

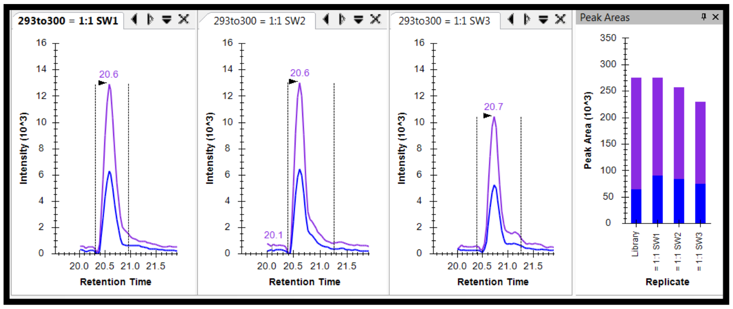

B SWATH Phospho-Isomer differentiation – ratios pS293/ pS300 + 4:1 and 1:4

YHGH**pS**MSDPGVSY**R**

pS-293

y6 - 688.3652+ (heavy)

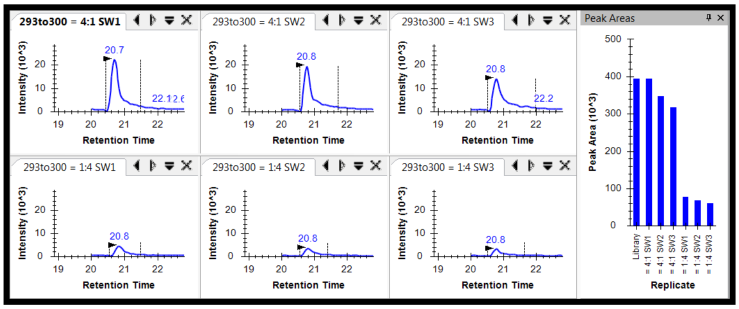

YHGHSMSDPGV**pS**YR

pS-300

y6 - 768.3316+ (heavy)
y6 -98 - 670.3547+ (heavy)

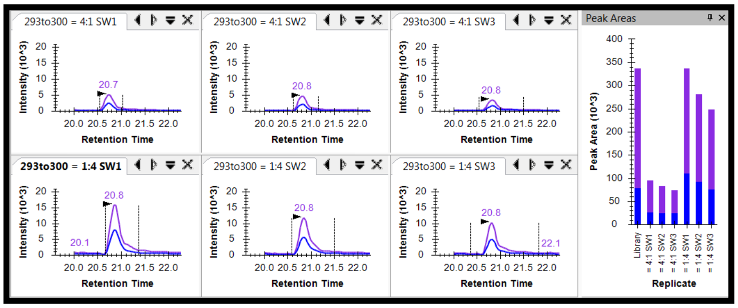

Supplemental Fig. S2

C SWATH Phospho-Isomer differentiation – ratios pS293/ pS300 + 8:1 and 1:8

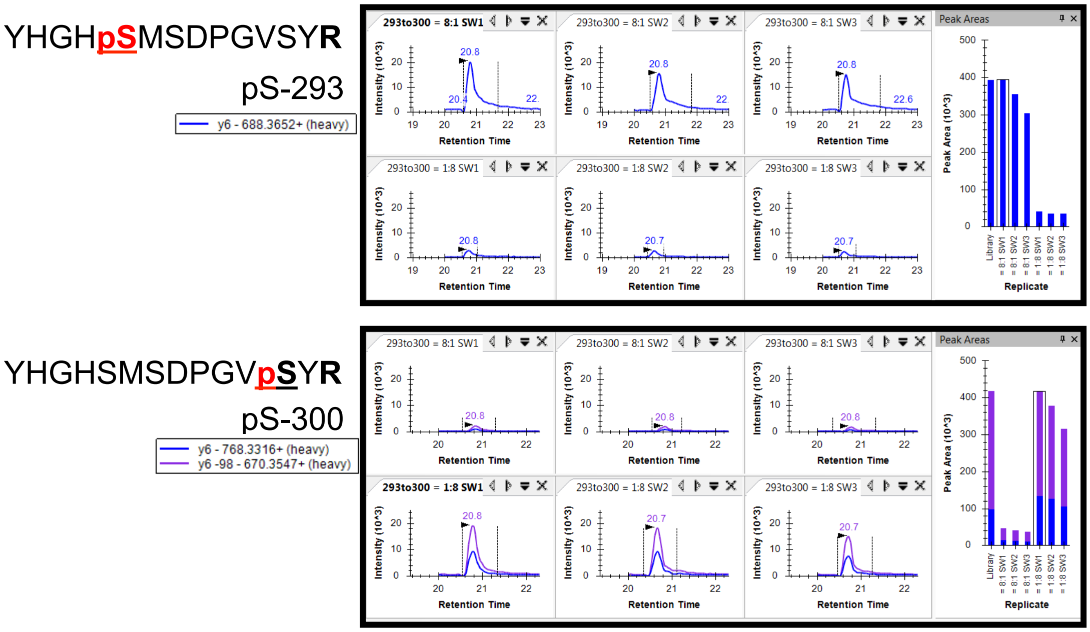

D SWATH Phospho-Isomer differentiation – ratios pS293/ pS300 + 16:1 and 1:16

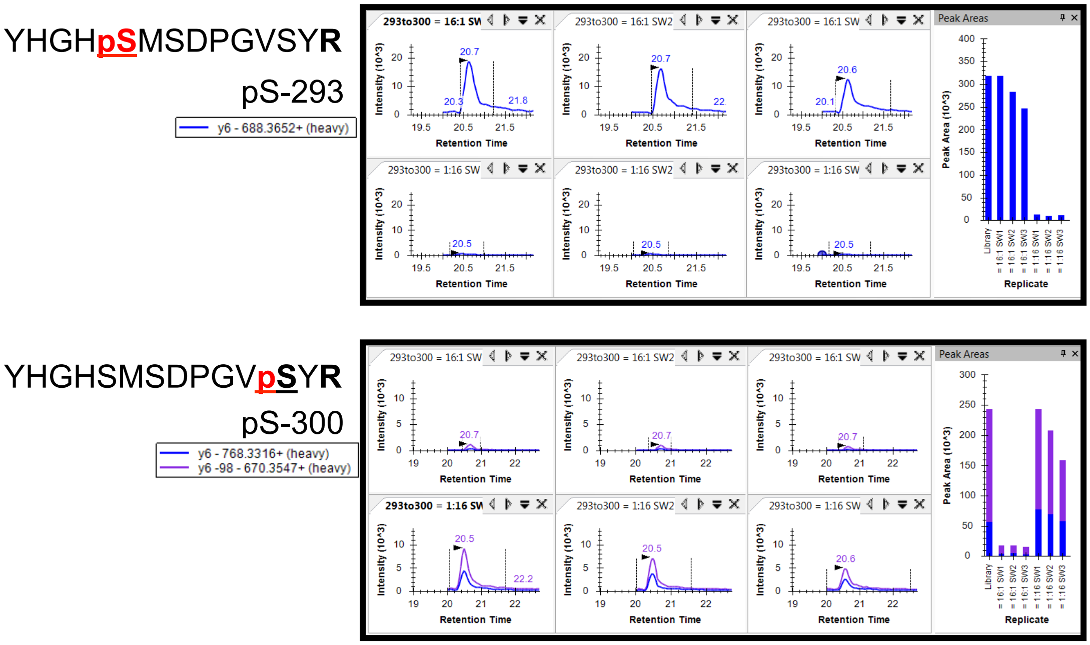

Supplemental Fig. S2. SWATH Phospho Isomer differentiation at different ratios across the isomer response curve. Isomers of heavy labeled mono phosphopeptides YHGHpS^{293}MSDPGVSYR and YHGHSMSDPGVpS^{300}YR were mixed at different concentration levels, all measurements were acquired in technical replicates. *A*, ratio pS293/pS300 = 1:1; *B*, ratio pS293/pS300 = 4:1 and 1:4; *C*, ratio pS293/pS300 = 8:1 and 1:8; and *D*, ratio pS293/pS300 = 16:1 and 1:16.
