# Supplementary material for: MS1 Peptide Ion Intensity Chromatograms in MS2 (SWATH) Data Independent Acquisitions. Improving Post Acquisition Analysis of Proteomic Experiments
Source: Mol Cell Proteomics. 2015 May 17;14(9):2405–19. doi: 10.1074/mcp.O115.048181 (PMC4563724; doi:10.1074/mcp.O115.048181)
Supplement: Supplemental Data [file supp_O115.048181_mcp.O115.048181-5.pdf]

Supplemental Fig. S3

SWATH reproducibility for MS2 fragment ions (80 peptides)

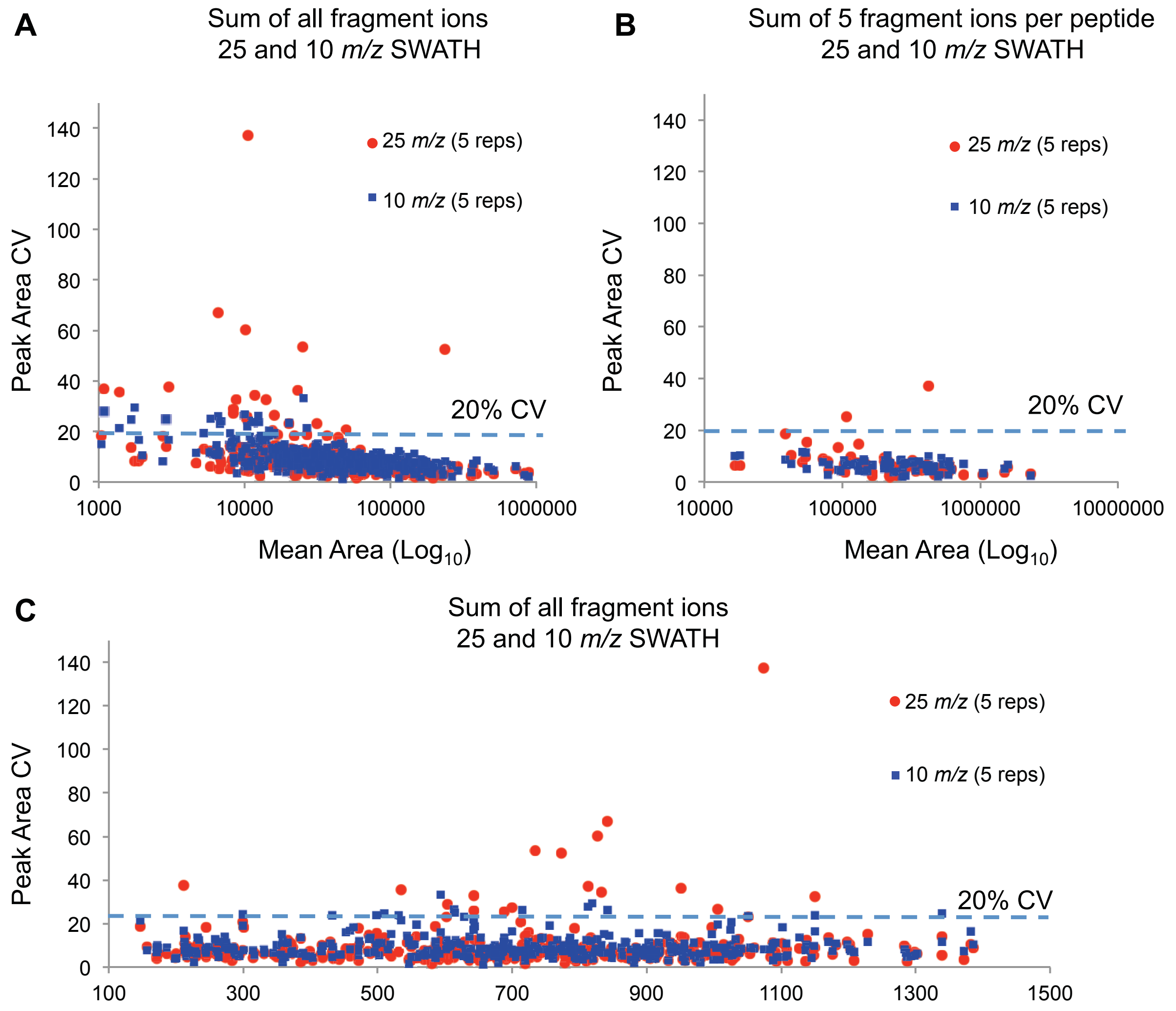

Supplemental Fig. S3. Mitochondrial lysate was analyzed by SWATH acquisition on a TripleTOF 5600 using SWATH segment window widths of 25 m/z and 10 m/z, respectively. Five replicates were acquired for each segment width with 300 ng sample loaded on column. 80 peptides were monitored by MS1 Filtering (precursor ion quantitation) and SWATH-MS2 Filtering (fragment ion quantitation, 5 fragment ions per peptide were monitored). Peak area CV's were calculated across 5 replicates each for 25 m/z and 10 m/z SWATH segments. A, scatter plots for all individual MS2 fragment ions plotting Peak Area CVs against Mean Peak Area for acquisitions with SWATH segment width 25 m/z (red) and 10 m/z (blue). B, Scatter plots for the sum of 5 fragment ions per peptide plotting Peak Area CVs against Mean Area for acquisitions with SWATH segment width 25 m/z (red) and 10 m/z (blue). C, Comparison of Scatter plots for all individual fragment ions plotting Peak Area CVs against fragment ion m/z for acquisitions with SWATH segment width 25 m/z (red) and 10 m/z (blue).
