# Supplementary material for: MS1 Peptide Ion Intensity Chromatograms in MS2 (SWATH) Data Independent Acquisitions. Improving Post Acquisition Analysis of Proteomic Experiments
Source: Mol Cell Proteomics. 2015 May 17;14(9):2405–19. doi: 10.1074/mcp.O115.048181 (PMC4563724; doi:10.1074/mcp.O115.048181)
Supplement: Supplemental Data [file supp_O115.048181_mcp.O115.048181-6.pdf]

Supplemental Fig. S4

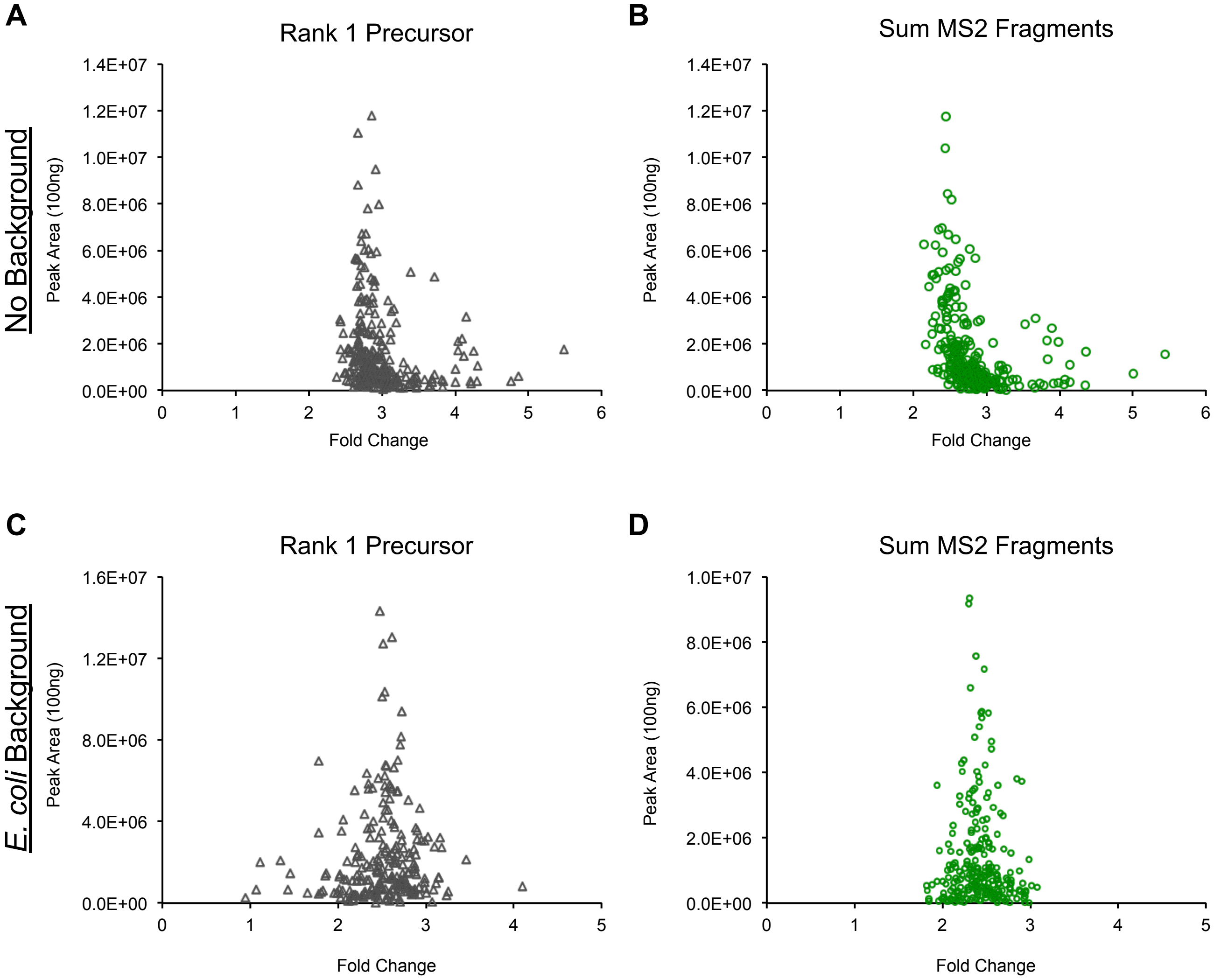

Supplemental Fig. S4. Comparison of peptide abundance in the 100 ng mitochondrial lysate to fold change after feature selection using IDSA. *A*, The rank 1 precursor peak area of the mitochondrial lysate at 100 ng and no background was plotted against the measured fold change (true fold change 3). *B*, The fragment ions for a given peptide precursor were summed and plotted against the measured fold change. *C*, The rank 1 precursor peak area of the mitochondrial lysate at 100 ng spiked into an *E. coli* background of 300 ng was plotted against the measured fold change (true fold change 3). *D*, The fragment ions for each peptide precursor were summed and plotted against the measured fold change from 100 ng mitochondrial lysate spiked into the *E. coli* background. The data supporting these figures can be found in Table S1*C* and S1*D*.
